# RS, S (+) - and R (−)-ibuprofen cocrystal polymorphs: Vibrational spectra, XRD measurement and DFT calculation studies

**DOI:** 10.1016/j.heliyon.2025.e41986

**Published:** 2025-01-20

**Authors:** Yaqi Jing, Qiuhui Zhao, Jiale Zhang, Jiadan Xue, Jianjun Liu, Jianyuan Qin, Zhi Hong, Yong Du

**Affiliations:** a Centre for THz Research, China Jiliang University, Hangzhou, 310018, China; b Department of Chemistry, Zhejiang Sci-Tech University, Hangzhou, 310018, China

**Keywords:** Chiral structure of ibuprofen, Nicotinamide, Cocrystal polymorphism, Vibrational spectra, XRD measurement, Structural optimization, DFT calculation

## Abstract

In this paper, cocrystal polymorphs of RS-ibuprofen (RS-IBU), S (+)-ibuprofen (S(+)-IBU), R (−)-ibuprofen (R(−)-IBU) with nicotinamide (NIC) were synthesized by different methods. RS-IBU is a chiral drug with only one chiral center in the molecule, which has two enantiomers (S (+)-IBU and R (−)-IBU). Due to the low solubility and bioavailability of IBU, its application is limited. The pharmaceutical cocrystal technology can improve the physicochemical properties of the drug. In this paper, we characterized RS-IBU, S (+)-IBU, R (−)-IBU, NIC, physical mixtures and cocrystal polymorphs by terahertz (THz), Raman and X-ray Diffraction (XRD), respectively. By observing the experimental results, we could clearly distinguish the cocrystal polymorphs. We found that the melt recrystallization method can generate cocrystal form A, while the solvent drop grinding method and solution evaporation method can generate cocrystal form B. In addition, in order to verify the successful preparation of them, we used density functional theory (DFT) to optimize and simulate the theoretical structures of the RS-IBU: NIC cocrystal polymorphs, and compared the simulated results with the experimental results. These research results provide a reference for the analysis and preparation of pharmaceutical cocrystal polymorphs and help to distinguish the cocrystal polymorphs.

## Highlights

• RS-IBU: NIC, S (+)-IBU: NIC and R (−)-IBU: NIC cocrystal polymorphs are prepared by melting recrystallization, solution evaporation crystallization and solvent drop grinding methods.
• The generated cocrystal polymorphs of chiral IBU: NIC are characterized by THz, Raman vibrational spectroscopy and XRD measurement.
• DFT simulations are performed on both structural optimization and frequency assignment of chiral IBU: NIC cocrystal polymorphs, and proving the successful preparation of such cocrystals.

## Graphical abstract

**Figure:**
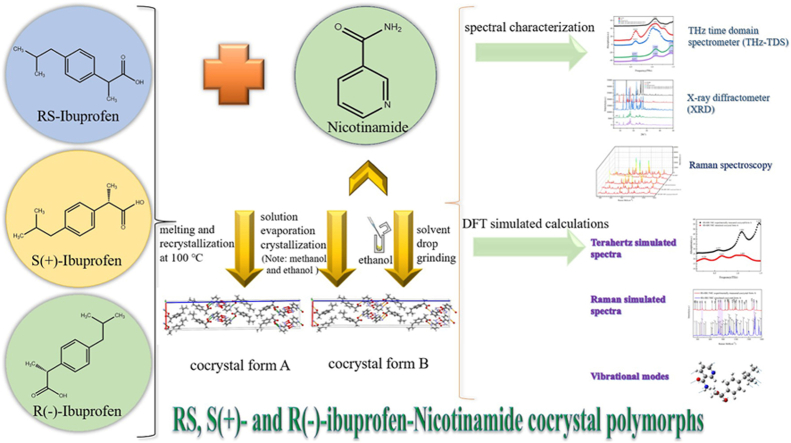

## 1 Introduction

Pharmaceutical cocrystals refer to crystals formed by combining active pharmaceutical ingredients (APIs) and cocrystal formers (CCFs) in a fixed stoichiometric ratio through hydrogen bonds or other non-covalent bonds. Nowadays, inflammation is an extremely common disease in people's daily lives. Due to defects in physical and chemical properties such as solubility and bioavailability, the clinical application of anti-inflammatory drugs can also be seriously affected [1]. Nonsteroidal anti-inflammatory drugs (NSAIDs) are a class of anti-inflammatory drugs. These drugs include ibuprofen, acetaminophen, indomethacin, ethylamine …, which have anti-inflammatory, analgesic, antipyretic and other effects, and are widely used clinically to relieve fever and various pain symptoms [2]. The efficacy of a drug usually depends on the physical and chemical properties of the APIs. The formation of drug cocrystals can improve the physical and chemical properties of the drugs, such as solubility, stability, bioavailability and dissolution rate, without changing the covalent structure of the drug molecule [3].

**Fig. 1:**
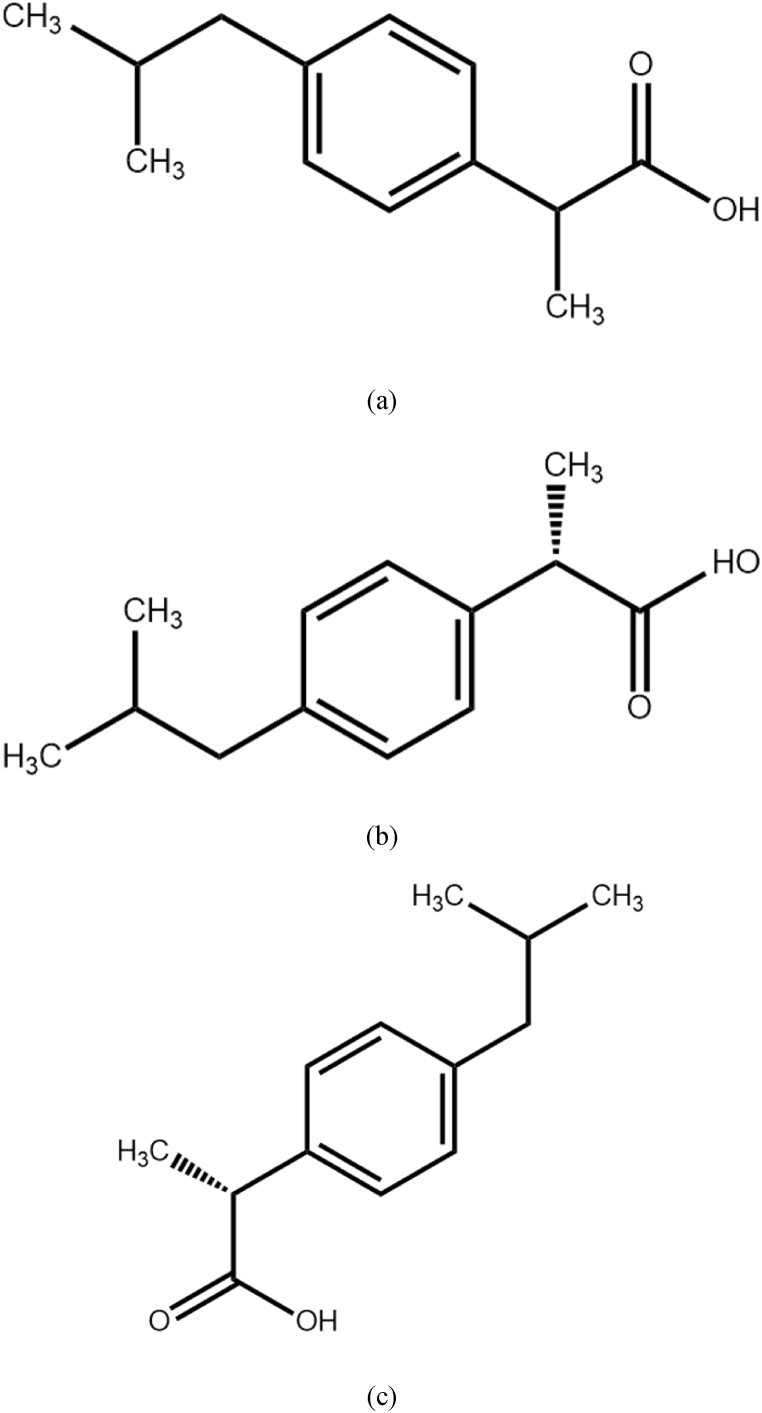
Molecular structures of RS-IBU (a), S (+)-IBU (b) and R (−)-IBU (c).

RS-IBU, whose molecular structure is shown in Fig. 1 (a) and whose chemical formula is C_13_H_18_O_2_, is a nonsteroidal anti-inflammatory drug that can be used to relieve mild to moderate pain and colds caused by fever [4]. At present, the development of chiral drugs has become one of the most important research contents in the pharmaceutical industry [5]. IBU exerts pharmacological activity by inhibiting the synthesis of prostaglandin (PG). S (+)-IBU is the main component of its pharmacological activity, while R (−)-IBU has little inhibitory effect on PG. Studies have shown that the activity of S (+)-IBU is 160 times that of R (−)-IBU and 1.6 times that of RS-IBU [6].

The molecular structures of S (+)-IBU and R (−)-IBU are shown in Fig. 1 (b) and 1 (c) respectively. In terms of molecular structure, there is a benzene ring and a carboxyl group in each IBU molecules.

**Fig. 2:**
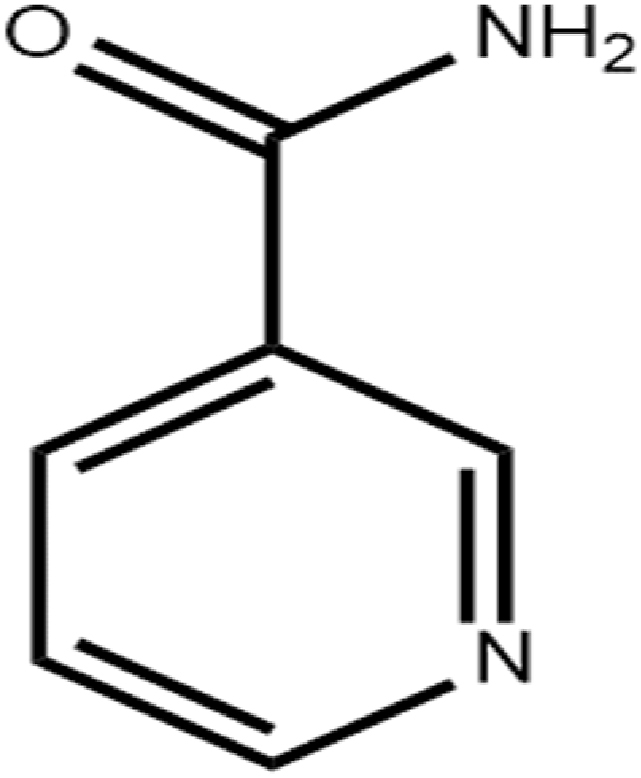
Molecular structure of NIC.

NIC, whose molecular structure is shown in Fig. 2 and whose chemical formula is C_6_H_6_N_2_O, is the amide form of vitamin B3 and is widely used in treating acne vulgaris, melasma, atopic dermatitis, rosacea, and preventing non-melanoma skin cancer. In addition, low doses of NIC have neuroprotective effects and can inhibit the development of diabetes [7]. It is a white crystalline powder that can be easily dissolved in water or ethanol [8]. In terms of molecular structure, there is an amide group and a pyridine ring in each NIC molecule [9]. The carboxyl group of the IBU molecule is located in the peripheral part of the assembly and can be connected to the nicotinamide dimer through O-H···N bonds. It is worth noting that intermolecular hydrogen-bonding interactions can also be formed between the amide groups of NIC molecules. Over the past two decades, a series of cocrystals between anti-inflammatory drugs and various CCFs have been reported, with NIC molecules often used as the CCF to form cocrystals [10]. In this paper, we will prepare and report the 1:1 cocrystal polymorphs of RS-IBU: NIC, S (+)-IBU: NIC, and R (−)-IBU: NIC using different synthetic methods.

DFT is developed based on the Maxwell-Schrödinger equation as well as external electromagnetic potential and internal magnetic field, which is often used in quantum chemical simulation calculation related research [11]. Some research groups have reported literatures on combining vibrational spectral techniques such as terahertz (THz) spectroscopy and Raman spectroscopy with DFT simulated calculations to study the interactions between and within drug cocrystal molecules. This method can determine the most reasonable molecular structure forms and vibration modes for preparing cocrystals [[12], [13], [14]]. Based on DFT, this paper combined Gaussian 16 and Gaussian-View software to perform structural optimization and simulated calculations on the theoretical structures of RS-IBU: NIC cocrystal form A and cocrystal form B [15,16].

Cambridge Crystallographic Data Centre (CCDC) is a database that specializes in collecting crystal structure information of organic and organometallic compounds. The database obtains crystal data through X-ray crystallography or neutron diffraction experiments, including the atomic coordinates, space group and unit cell parameters of the compounds. Berry et al. [17] prepared and reported the powder X-ray diffraction data of RS-IBU: NIC cocrystal form A by hot melt extrusion, and its CCDC reference number is 678915. Alshahateet et al. [18] prepared and reported the single crystal structure of RS-IBU: NIC cocrystal form B by solvent evaporation, and its CCDC reference number is 773196. In this paper, we synthesized RS-IBU: NIC, S (+)-IBU: NIC and R (−)-IBU: NIC cocrystal polymorphs by the same preparation methods. Based on this, this paper simulated and calculated the THz and Raman vibration spectra of the two cocrystal forms of RS-IBU: NIC. By comparing the simulation results with the experimental results, the intermolecular interactions between the two cocrystal forms were further analyzed and verified.

In this paper, the combination of various spectral detection techniques and DFT simulations provides data support for the analysis and preparation of pharmaceutical cocrystal polymorphs. We can use these results to distinguish RS-IBU: NIC, S (+)-IBU: NIC and R (−)-IBU: NIC cocrystal polymorphs.

## 2 Experimental and theoretical section

### 2.1 Sample preparation

RS-IBU (purity ≥98 %), S (+)-IBU (purity ≥98.5 %), R (−)-IBU (purity ≥98 %), NIC (purity ≥99.5 %), methanol (purity ≥99.9 %) and ethanol (purity ≥98 %) were purchased from J&K Scientific Ltd (Shanghai, China) and Mick Chemical Instrument Co., Ltd. (Hangzhou, China). All pharmaceutical products were used without further purification.

#### 2.1.1 Preparation of two cocrystal forms of RS-IBU: NIC

(1) Cocrystal form A

Melting recrystallization method: Weigh RS-IBU (206.27 mg, 1 mmol) and NIC (122.13 mg, 1 mmol), put them into a conical centrifuge tube at a stoichiometric ratio of 1:1, and place the conical centrifuge tube in a vortex mixer to rotate up for about 2 min, place the fully mixed physical mixture in a glass evaporating dish, then heat it to 100 °C to melt in a vacuum drying oven, and finally cool it at room temperature for recrystallization. After about 24 h, white massive crystals were obtained.

(1) Cocrystal form B
1) Solution evaporation crystallization method

Weigh RS-IBU (206.27 mg, 1 mmol) and NIC (122.13 mg, 1 mmol) and place them in a glass evaporating dish, then dissolve them in 3 mL methanol at a stoichiometric ratio of 1:1, and slowly evaporate and crystallize at room temperature. After about 24 h, white massive crystals were obtained.

Weigh RS-IBU (206.27 mg, 1 mmol) and NIC (122.13 mg, 1 mmol) and place them in a glass evaporating dish, then dissolve them in 10 mL ethanol at a stoichiometric ratio of 1:1, and slowly evaporate to crystallize at room temperature. After about 24 h, white massive crystals were obtained.

2) Solvent drop grinding method

Weigh RS-IBU (618.8 mg, 3 mmol) and NIC (366.4 mg, 3 mmol) and place them in a 25 mL stainless steel grinding jar. Use a planetary ball mill to grind with a 1:1 stoichiometric ratio. Before the grinding starts, add 0.5 mL of free water ethanol, and then ground at room temperature for 1 h at a frequency of 30 Hz. After grinding, dried at room temperature and obtained white massive crystals after about 2 days.

#### 2.1.2 Preparation of two cocrystal forms of S (+)-IBU: NIC

(1) Cocrystal form A

The preparation method is the same as that of RS-IBU: NIC cocrystal form A (heating it to 90 °C in a vacuum drying oven to melt).

(2) Cocrystal form B

The preparation method was the same as that of RS-IBU: NIC cocrystal form B.

#### 2.1.3 Preparation of two cocrystal forms of R (−)-IBU: NIC

(1) Cocrystal form A

The preparation method is the same as that of RS-IBU: NIC cocrystal form A (heating it to 85 °C in a vacuum drying oven to melt).

(2) Cocrystal form B

The preparation method was the same as that of RS-IBU: NIC cocrystal form B.

#### 2.1.4 Physical mixture preparation and subsequent processing

Physical mixture: Weigh RS-IBU (206.27 mg, 1 mmol)/S (+)-IBU (206.27 mg, 1 mmol)/R (−)-IBU (206.27 mg, 1 mmol) and NIC (122.13 mg, 1 mmol) respectively. Put them into a conical centrifuge tube at a stoichiometric ratio of 1:1, and placed the conical centrifuge tube on a vortex mixer to rotate. After about 2 min, uniform mixtures were obtained, respectively. In this paper, we prepared physical mixtures to prove that the cocrystal form A and form B prepared by the three kinds of IBU molecules with NIC molecules were not simply mixed by the starting components. We introduced the preparation methods of physical mixtures and cocrystal polymorphs, and experimentally proved that the formation of cocrystal polymorphs has nothing to do with physical mixing using various spectral characterization methods.

Grind RS-IBU, S (+)-IBU, R (−)-IBU, NIC, their respective physical mixtures, cocrystal form A and cocrystal form B into powder and put them into a tablet press at a pressure of 6 MPa, leaving on for about 1 min. The samples were prepared into a disc with a diameter of about 13 mm and a thickness of 1∼2 mm, and then placed in a sealed bag for subsequent THz detection. For Raman and XRD detection, only a small amount of powder sample was needed, and no further tableting was required to prepare the sample.

### 2.2 A solubility test experiment

Our existing experimental conditions are not sufficient to support our solubility test experiments. By consulting literatures, we found that Yuliandra et al. [19] conducted a solubility test experiment on RS-IBU: NIC cocrystal form B prepared by solution evaporation crystallization. The results showed that the formation of cocrystals significantly enhanced the solubility of RS-IBU compared with the physical mixture and pure RS-IBU. Specifically, the solubility of the cocrystal is about 1.5 times that of pure RS-IBU and approximately 1.2 times that of the physical mixture.

### 2.3 Vibrational spectra and XRD measurement

#### 2.3.1 THz spectra

We used the Z2 (Zomega Company of the United States) transmission measurement system to measure the THz spectra of three APIs, NIC, corresponding physical mixtures and cocrystals. The device used a light pulse sequence from a mode-locked Ti: sapphire laser system (Spectral Physics, USA) to drive a photoconductive switch to generate and detect terahertz radiation. The frequency used is 75 MHz, the pulse width is 100 fs, and the center wavelength is 780 nm [20]. All samples were measured at room temperature, in a dry, nitrogen atmosphere with a relative humidity of less than 1 %. Terahertz Time Domain Spectroscopy (THz-TDS) is a spectral technique that uses short pulses of THz radiation to study the properties of matter. This technique is very sensitive to sample effects and can detect amplitude and phase changes of THz radiation. The samples were placed in the THz-TDS system, and nitrogen was continuously blown into the terahertz path to reduce the absorption of environmental moisture by radiation and ensure the accuracy of experimental data. The frequency response of the samples was divided by the frequency response of the reference sample by fast Fourier transform (FFT) to obtain the terahertz absorption spectra.

#### 2.3.2 Raman spectra

In this work, a Nicolet Raman 960 Fourier transform Raman spectrometer (Thermo Nicolet, Madison, USA) and a diode-pumped solid-state laser (wavelength 1064 nm) were used as near-infrared light sources to obtain the Raman spectra of three APIs, NIC, corresponding physical mixtures, and cocrystals. The Raman spectra range used in the sample is 200∼1800 cm^−1^, the laser radiation is 785 nm, the spectral resolution is 2 cm^−1^, and the laser power is 150 mW.

#### 2.3.3 XRD measurement

SmartLab SE X-ray diffractometer (Rigaku Corporation, Japan) was used to measure the XRD patterns of three APIs, NIC, corresponding physical mixtures and cocrystals. The principle of XRD is based on Bragg's law, that is, when X-rays pass through a crystal, diffraction will occur. The diffraction angle is related to the crystal structure and is the main method for quantification of polymorphs [21]. XRD can be used to identify and determine the physical phase of ordinary powder samples, pharmaceuticals and film samples, etc. [22]. Among them, the minimum step size used in the test sample in this article is θ s: 0.0001°, θ d: 0.0001°, and the selected 2θ range is 5∼50°.

### 2.4 DFT theoretical calculation

As a powerful tool for quantum chemical simulation, Gaussian software can be used for geometric optimization of various chemical structures and simulation calculations of vibration frequencies [23,24]. In this article, we used the Becke-3-Lee-Yang-Parr (B3LYP) functional and the 6–311++g(d,p) basis set, combined with Gaussian 16 and Gaussian-View software, to analyze RS-IBU: NIC at the DFT level [25,26]. The molecular structures of cocrystal form A and RS-IBU: NIC cocrystal form B were optimized and simulated to further analyze the intermolecular interactions of the two cocrystal forms. By analyzing the formation process of drug cocrystals through THz spectroscopy and Raman vibrational spectroscopy technology combined with DFT simulation results, the intermolecular and intramolecular interactions of RS-IBU: NIC cocrystals could be effectively analyzed, which was useful for understanding the structural characteristics and vibration modes of the sample [27]. In the DFT calculations at the B3LYP level, we convolved the Lorentz lines into the calculated vibrations using a full width at half maximum (FWHM) of 4.0 cm^−1^.

## 3 Results and discussion

### 3.1 Comparison of crystal structures of RS-IBU: NIC cocrystal polymorphs

**Fig. 3:**
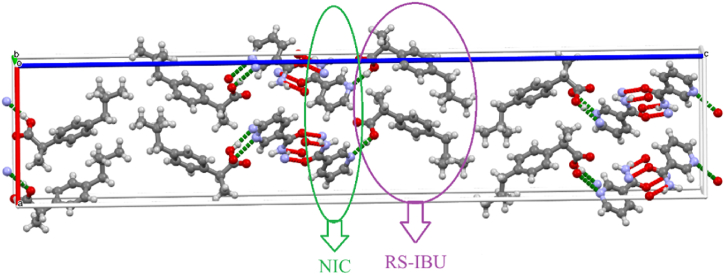
Crystal structure diagram of RS-IBU: NIC cocrystal form A. (Note: The red dotted line represents the intermolecular hydrogen-bonding interactions between NIC molecules; the green dotted line represents the intermolecular hydrogen-bonding interactions between RS-IBU and NIC molecules).

**Fig. 4:**
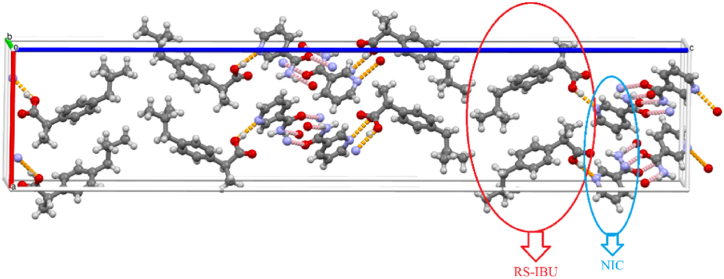
Crystal structure diagram of RS-IBU: NIC cocrystal form B. (Note: The pink dotted line represents the intermolecular hydrogen-bonding interactions between NIC molecules; the orange dotted line represents the intermolecular hydrogen-bonding interactions between RS-IBU and NIC molecules).

The crystal structures of RS-IBU: NIC cocrystal form A and RS-IBU: NIC cocrystal form B are both from the CCDC, and their CCDC reference numbers are 678915 and 773196 respectively [17,18]. The crystal structure of RS-IBU: NIC cocrystal form A was shown in Fig. 3. In Fig. 3, the green ellipse marks NIC molecules, and the purple ellipse marks RS-IBU molecules. The crystal structure of RS-IBU: NIC cocrystal form A belongs to the orthorhombic system. RS-IBU: NIC cocrystal form A generates O_21_-H_21_···N_52_, O_41_-H_41_···N_51_ and O_1_-H_1_···N_42_ intermolecular hydrogen-bonding interactions through the hydroxyl group in the RS-IBU molecules and the N atoms on the pyridine ring of the NIC molecules. In addition, the H atoms of the imino group in one NIC molecule and the O atoms of the amide group in another NIC molecule are connected through N_51_-H_51A_···O_41_ and N_41_-H_41A_···O_51_ intermolecular hydrogen-bonding interactions respectively, forming a four-ring unit structure. The crystal structure of RS-IBU: NIC cocrystal form B was shown in Fig. 4. In Fig. 4, the red ellipse marks RS-IBU molecules, and the blue ellipse marks NIC molecules. The crystal structure of RS-IBU: NIC cocrystal form B belongs to the orthorhombic system. RS-IBU: NIC cocrystal form B generates O_6_-H_6A_···N_2_ and O_4_-H_4A_···N_4_ intermolecular hydrogen bonds through the hydroxyl group in the RS-IBU molecules and the N atoms on the pyridine ring of the NIC molecules. In addition, the O atoms of the amide group in one NIC molecule and the H atoms of the imino group in another NIC molecule form an O_2_···H_1A_-N_1_ intermolecular hydrogen-bonding interaction. The H atoms of the imino group in one NIC molecule and the O atoms of the amide group in another NIC molecule forms N_3_-H_3A_···O_1_ intermolecular hydrogen-bonding interaction.

Since we are unable to perform single crystal X-ray diffraction experiments, we are temporarily unable to obtain the crystal structure information of S (+)-IBU: NIC and R (−)-IBU: NIC cocrystal polymorphs.

In order to better describe the differences in the crystal structures of RS-IBU: NIC cocrystal form A and RS-IBU: NIC cocrystal form B, we tabulated some crystallographic data of the two cocrystal forms in Supporting Information, as shown in Table S1.

Checking the CCDC, it could be found that there were very few studies on the single crystal structures of S (+)-IBU: NIC and R (−)-IBU: NIC cocrystal polymorphs, and there was no relevant reference numbers generated. By comparing the crystal structures of RS-IBU: NIC cocrystal form A and RS-IBU: NIC cocrystal form B, it can be found that the hydrogen-bonding elements between RS-IBU and NIC molecules in the two cocrystal forms are the same. There are differences in the hydrogen-bonding components between NIC molecules, which may lead to the generation of cocrystal polymorphs. Therefore, we hypothesized that the cocrystal polymorphs of S (+)-IBU: NIC and R (−)-IBU: NIC could be generated by the same preparation method. This article will next use vibrational spectroscopy experiments to verify the existence of RS-IBU: NIC, S (+)-IBU: NIC and R (−)-IBU: NIC cocrystal polymorphs.

Molecular docking is the study of intermolecular interactions (such as ligands and receptors). In this paper, we analyzed the intermolecular hydrogen-bonding interactions of RS-IBU: NIC cocrystal polymorphs. By comparing the crystal structures of RS-IBU: NIC cocrystal form A and RS-IBU: NIC cocrystal form B, it can be found that the hydrogen-bonding heterogeneous elements between RS-IBU and NIC molecules in the two cocrystal crystal forms are the same, while the hydrogen-bonding heterogeneous elements between NIC molecules are different, which just shows that it is the different intermolecular hydrogen-bonding interactions that lead to the emergence of cocrystal polymorphism. Therefore, the study of molecular docking plays a pivotal role in this work.

### 3.2 Terahertz spectral characterization and analysis of RS-IBU: NIC, S (+)-IBU: NIC and R (−)-IBU: NIC cocrystal polymorphs

THz-TDS, as a universal fingerprint spectroscopy technology involving molecular structures, can effectively analyze and identify subtle structural changes caused by intermolecular interactions [[27], [28], [29], [30]]. When considering energy level transitions, the energy lost by the drug co-crystal is consistent with the photon energy data of the THz wave itself. Therefore, the fingerprint characteristics of THz-TDS can be used to determine the spectral information and intermolecular hydrogen-bonding interactions of drug cocrystals through the vibration position of the spectra. The data of each sample were recorded in the time domain of the terahertz electric field, and the frequency response of the samples was divided by the frequency response of the reference sample through FFT to obtain the terahertz absorption spectra [31].

#### 3.2.1 Terahertz experimental spectra of RS-IBU: NIC cocrystal polymorphs

**Fig. 5:**
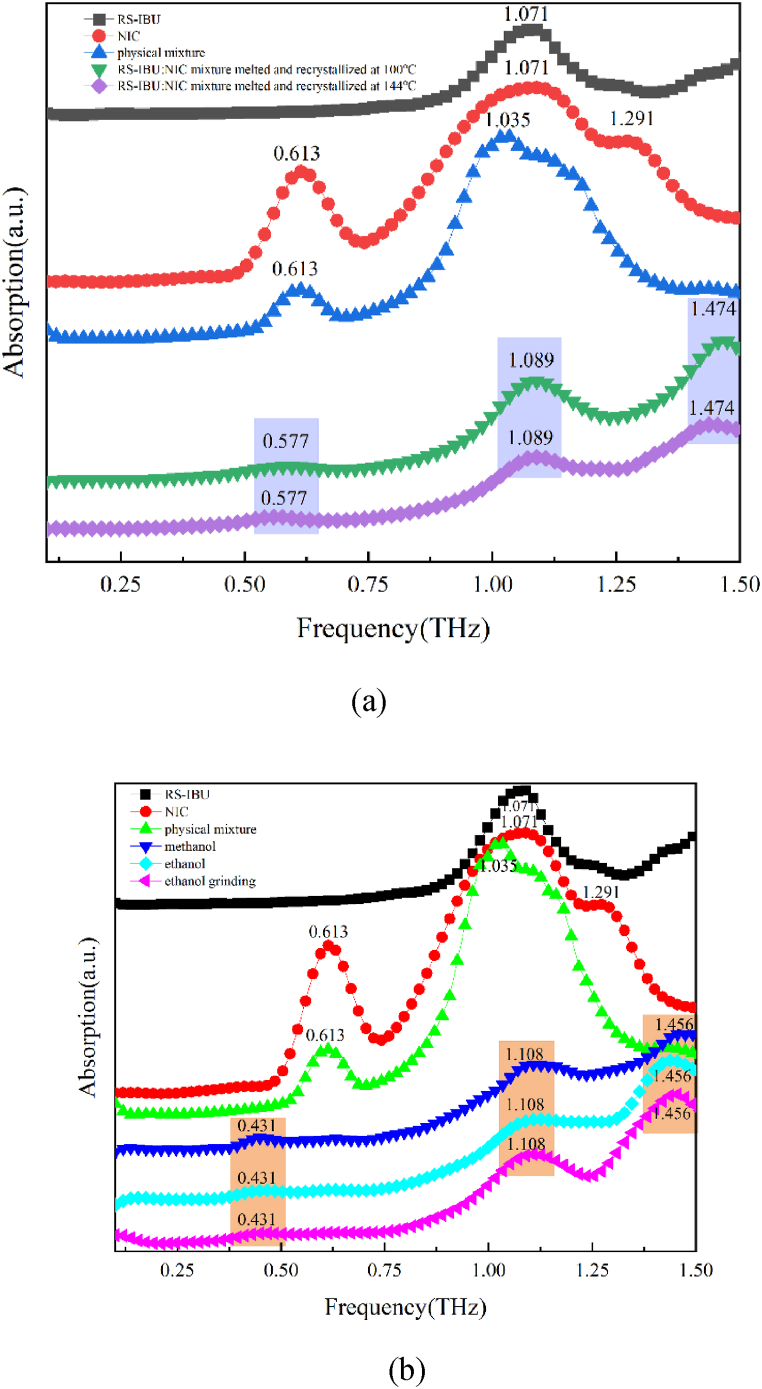
RS-IBU, NIC, physical mixture and RS-IBU: NIC cocrystal form A (a) RS-IBU, NIC, physical mixture and RS-IBU: NIC cocrystal form B (b) in the range of 0.1–1.5 THz spectra.

The THz absorption spectra of RS-IBU, NIC, physical mixture, RS-IBU: NIC cocrystal form A and RS-IBU: NIC cocrystal form B in the frequency range of 0.1–1.5 THz were shown in Fig. 5. As shown in Fig. 5 (a), the RS-IBU: NIC cocrystal form A has three obvious characteristic peaks at 0.577 THz, 1.089 THz and 1.474 THz. Ishihara et al. [32] found that RS-IBU: NIC cocrystal could be generated by melting crystallization. First, RS-IBU melted when it reached about 74 °C, and then the melted liquid RS-IBU and NIC (the melting point of NIC is about 130 °C) intermolecular interactions lead to the formation of RS-IBU: NIC cocrystal form A. We found through experiments that three characteristic peaks of 0.577 THz, 1.089 THz and 1.474 THz appeared at 100 °C and 144 °C (above 100 °C), which showed that RS-IBU: NIC cocrystal form A could be generated when heated to 100 °C, and the cocrystal form did not change as the temperature increased. In addition, in the range of 0.1–1.5 THz, IBU molecules only have an absorption peak at 1.071 THz, which was consistent with what Wang et al. [33] reported. In the range of 0.1–1.5 THz, NIC molecules have three absorption peaks, located at 0.613 THz, 1.071 THz and 1.291 THz respectively. The first two peaks were consistent with those reported by Zhang et al. [9]. The physical mixture of RS-IBU and NIC has absorption peaks at 0.613 THz and 1.035 THz, which showed that the mixture was a linear superposition of the starting components RS-IBU molecules and NIC molecules. Through comparison, it could be found that the absorption peaks of RS-IBU: NIC cocrystal form A were not observed in the starting components, and were completely different from the substance mixture. This might be caused by the intermolecular hydrogen-bonding interactions of RS-IBU and NIC, as well as the intermolecular hydrogen-bonding interactions of NIC.

As shown in Fig. 5 (b), the RS-IBU: NIC cocrystal form B has three obvious characteristic peaks at 0.431 THz, 1.108 THz and 1.456 THz. Alshahateet et al. [18] found that RS-IBU: NIC cocrystal form B could be generated through solvent evaporation crystallization. We found through experiments that three characteristic peaks of 0.431 THz, 1.108 THz and 1.456 THz appeared for RS-IBU and NIC through methanol solvent evaporation crystallization, ethanol solvent evaporation crystallization and ethanol solvent drop grinding methods, which showed that all three methods could generate RS-IBU: NIC cocrystal form B. In addition, through comparison, it could be found that the absorption peaks of RS-IBU: NIC cocrystal form B were not observed in the starting components and were completely different from the substance mixture. This might be due to the intermolecular hydrogen-bonding interactions formed between RS-IBU and NIC, as well as the O···H-N hydrogen-bonding interactions formed between NIC molecules.

Comparing the THz spectra of RS-IBU: NIC cocrystal form A and RS-IBU: NIC cocrystal form B, it could be found that their absorption peaks were completely different. There is a different hydrogen bond between the NIC molecules of RS-IBU: NIC cocrystal form A and cocrystal form B, which shows that there are differences in the crystal structures of them, and THz spectroscopy can detect this difference. These results can prove the successful preparation of two cocrystal forms of RS-IBU: NIC.

#### 3.2.2 Terahertz experimental spectra of S (+)-IBU: NIC cocrystal polymorphs

**Fig. 6:**
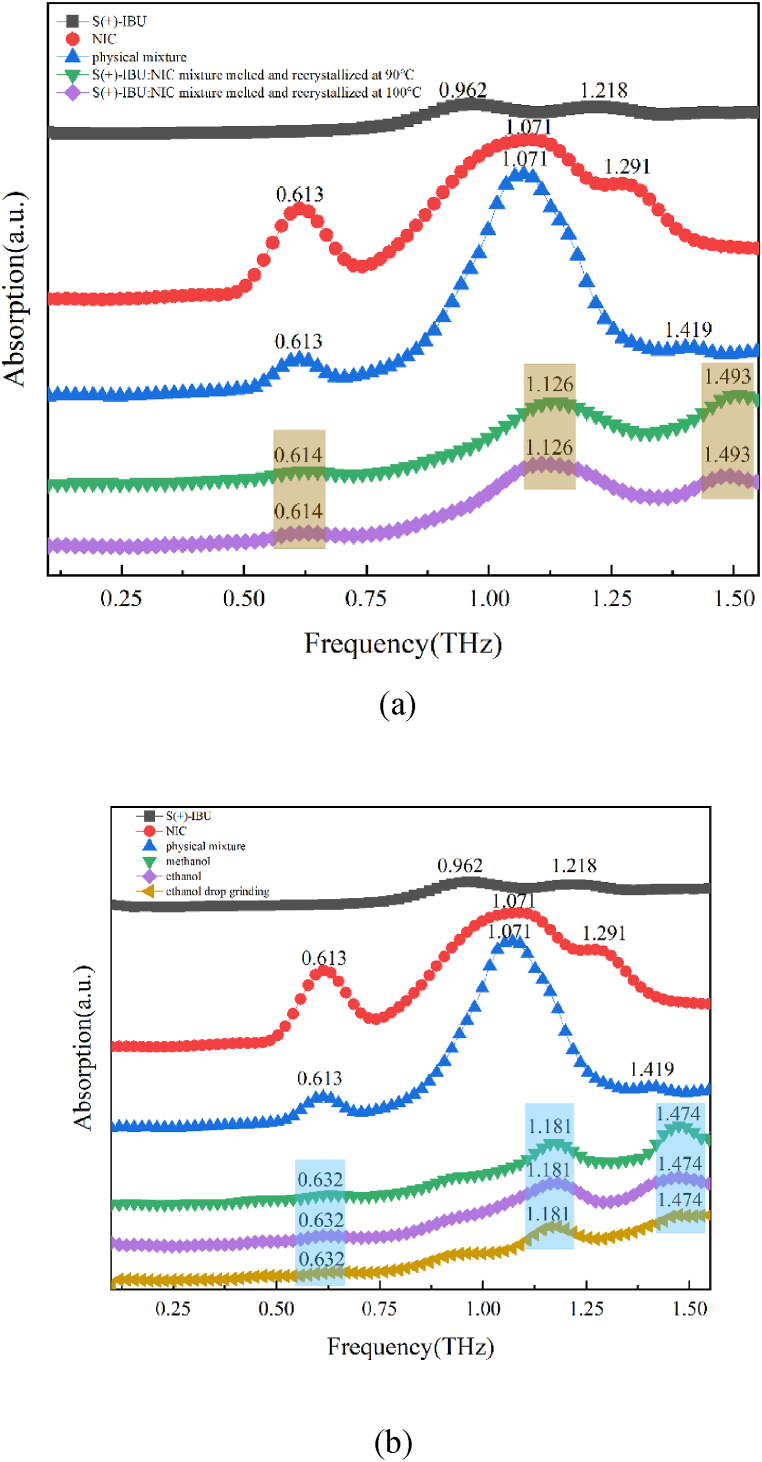
S (+)-IBU, NIC, physical mixture and S (+)-IBU: NIC cocrystal form A (a) S (+)-IBU, NIC, physical mixture and S (+)-IBU: NIC cocrystal form B (b) in the range of 0.1–1.5 THz spectra.

The THz absorption spectra of S (+)-IBU, NIC, physical mixture, S (+)-IBU: NIC cocrystal form A and S (+)-IBU: NIC cocrystal form B in the range of 0.1–1.5 THz were shown in Fig. 6. As shown in Fig. 6 (a), the S (+)-IBU: NIC cocrystal form A has three obvious characteristic peaks at 0.614 THz, 1.126 THz, and 1.493 THz. The melting point of RS-IBU is about 75 °C, and the melting point of S (+)-IBU is about 50 °C [34]. Therefore, we hypothesized that the formation temperature of S (+)-IBU: NIC cocrystal form A was lower than that of RS-IBU: NIC cocrystal form A. We found through experiments that three characteristic peaks of 0.614 THz, 1.126 THz and 1.493 THz appeared at 90 °C and 100 °C (above 90 °C), which showed that RS-IBU: NIC cocrystal form A could be generated when heated to 90 °C, and the cocrystal form did not change as the temperature increased. In addition, in the range of 0.1–1.5 THz, S (+)-IBU molecules have two absorption peaks at 0.962 THz and 1.218 THz. The physical mixture of S (+)-IBU and NIC has absorption peaks at 0.613 THz, 1.071 THz and 1.419 THz. The absorption peaks of S (+)-IBU: NIC cocrystal form A were not observed in the starting components. This showed that the mixture was a linear superposition of the starting components, and the absorption peaks of the cocrystal were not simply a linear superposition.

As shown in Fig. 6 (b), the S (+)-IBU: NIC cocrystal form B has three obvious characteristic peaks at 0.632 THz, 1.181 THz, and 1.474 THz. We found through experiments that the same three characteristic peaks appeared for S (+)-IBU and NIC through methanol solvent evaporation crystallization, ethanol solvent evaporation crystallization and ethanol solvent drop milling method, which showed that all three methods could generate S (+)-IBU: NIC cocrystal form B. In addition, through comparison, it could be found that the absorption peaks of S (+)-IBU: NIC cocrystal form B were not observed in the starting components and were completely different from the substance mixture.

Comparing the THz spectra of S (+)-IBU: NIC cocrystal form A and S (+)-IBU: NIC cocrystal form B, it could be found that their absorption peaks were completely different, and the THz spectrum could effectively identify molecules composed of subtle structural changes caused by interactions. These results can prove the successful preparation of two cocrystal forms of S (+)-IBU: NIC.

#### 3.2.3 Terahertz experimental spectra of R (−)-IBU: NIC cocrystal polymorphs

**Fig. 7:**
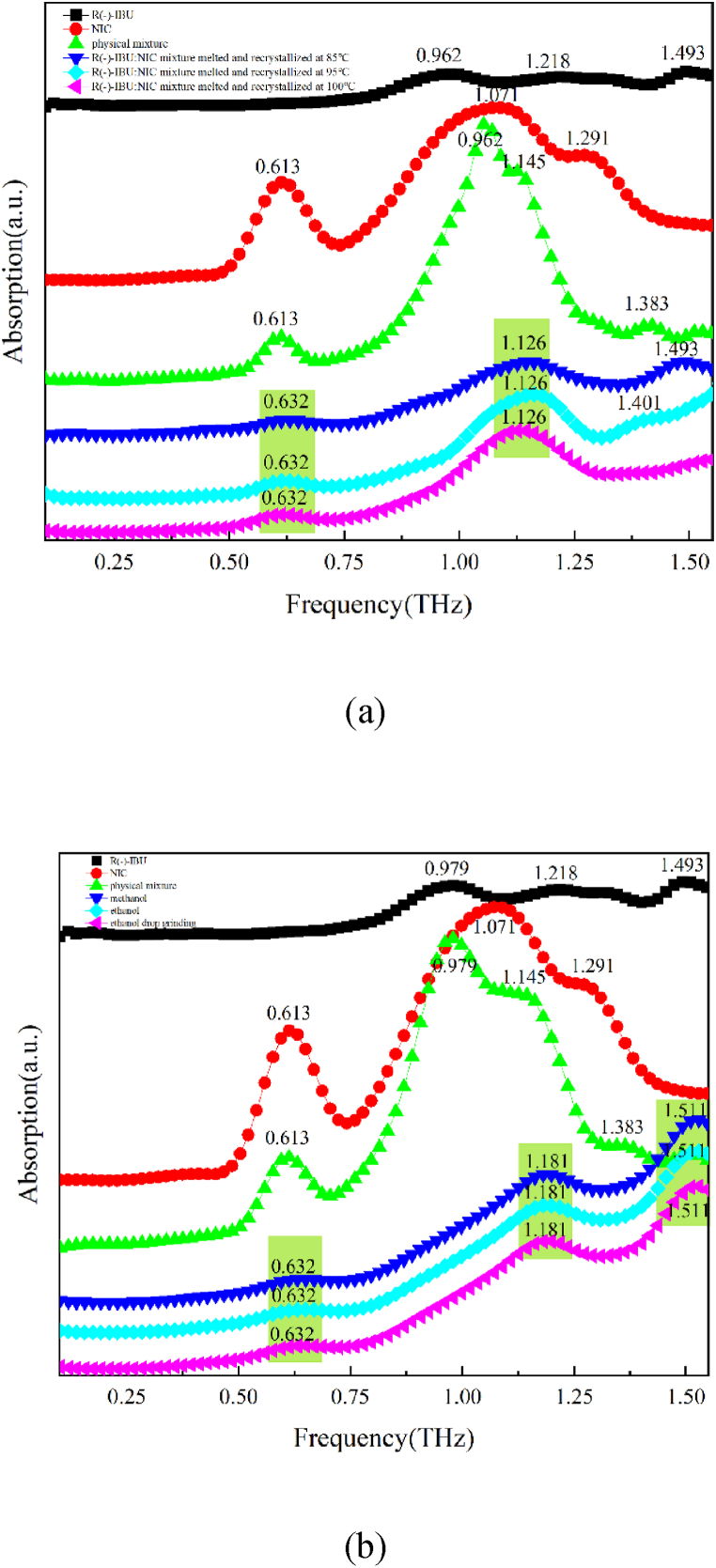
R (−)-IBU, NIC, physical mixture and R (−)-IBU: NIC cocrystal form A (a) R (−)-IBU, NIC, physical mixture and R (−)-IBU: NIC cocrystal form B (b) in the range of 0.1–1.5 THz spectra.

The THz absorption spectra of R (−)-IBU, NIC, physical mixture, R (−)-IBU: NIC cocrystal form A and R (−)-IBU: NIC cocrystal form B in the frequency range of 0.1–1.5 THz were shown in Fig. 7. As shown in Fig. 7 (a), R (−)-IBU: NIC cocrystal form A has two characteristic peaks at 0.632 THz and 1.126 THz. The melting point of R (−)-IBU is about 40 °C, which is lower than the melting point of RS-IBU and S (+)-IBU. Therefore, we guess that the formation temperature of R (−)-IBU: NIC cocrystal form A is lower than that of RS-IBU: NIC and S (+)-IBU: NIC cocrystal form A. Through experiments, we found that the same absorption peak as the S (+)-IBU: NIC cocrystal form A appeared at 85 °C. This might be due to the similar chemical properties of left- and right-handed drug molecules. As the heating temperature increased, the third absorption peak (1.493 THz) appeared red-shifted until it disappeared. At 100 °C, there were only two characteristic peaks at 0.632 THz and 1.126 THz. This showed that R (−)-IBU: NIC could be generated when heated to 100 °C, and the cocrystal form did not change as the temperature increased. In addition, in the range of 0.1–1.5 THz, R (−)-IBU molecules have three absorption peaks at 0.962 THz, 1.218 THz and 1.493 THz, among which the first two absorption peaks are consistent with the absorption peaks of S (+)-IBU molecules. The physical mixture of R (−)-IBU and NIC has absorption peaks at 0.613 THz, 0.962 THz, 1.145 THz and 1.383 THz. The absorption peaks of R (−)-IBU: NIC cocrystal form A were all not observed in the starting components, which showed that the mixture was a linear superposition of the starting components, and the absorption peak of the cocrystal was not a simple linear superposition.

As shown in Fig. 7 (b), R (−)-IBU: NIC cocrystal form B has three obvious characteristic peaks at 0.632 THz, 1.181 THz and 1.511 THz, among which the first two characteristic peaks are consistent with S (+)-IBU: NIC cocrystal form B. We found through experiments that the same three characteristic peaks appeared for R (−)-IBU and NIC through methanol solvent evaporation crystallization, ethanol solvent evaporation crystallization and ethanol solvent drop milling methods, which showed that all three methods could generate R (−)-IBU: NIC cocrystal form B.

Comparing the THz spectra of R (−)-IBU: NIC cocrystal form A and R (−)-IBU: NIC cocrystal form B, it could be found that only the first absorption peak was the same, and the last two absorption peaks were completely different, which shows that there are subtle differences in the crystal structures of the two, and THz spectroscopy can effectively identify such subtle changes. These results can prove the successful preparation of two cocrystal forms of R (−)-IBU: NIC.

### 3.3 Raman spectral characterization and analysis of RS-IBU: NIC, S (+)-IBU: NIC and R (−)-IBU: NIC cocrystal polymorphs

Raman spectroscopy technology studies the rotation or vibration of molecules based on the principle of spectral scattering, and can obtain vibration energy transition information of internal groups of molecules, which can be a good supplementary method to THz [35,36]. Raman spectroscopy technology based on light scattering can provide rich structural information about specific molecules, such as vibrational transitions of molecular and electronic polarization and vibrations of different internal groups of molecules. However, Raman spectroscopy focuses on the characterization of the vibrations of functional groups within molecules and cannot sensitively characterize intermolecular interactions.

#### 3.3.1 Raman experimental spectra of RS-IBU: NIC cocrystal polymorphs

**Fig. 8:**
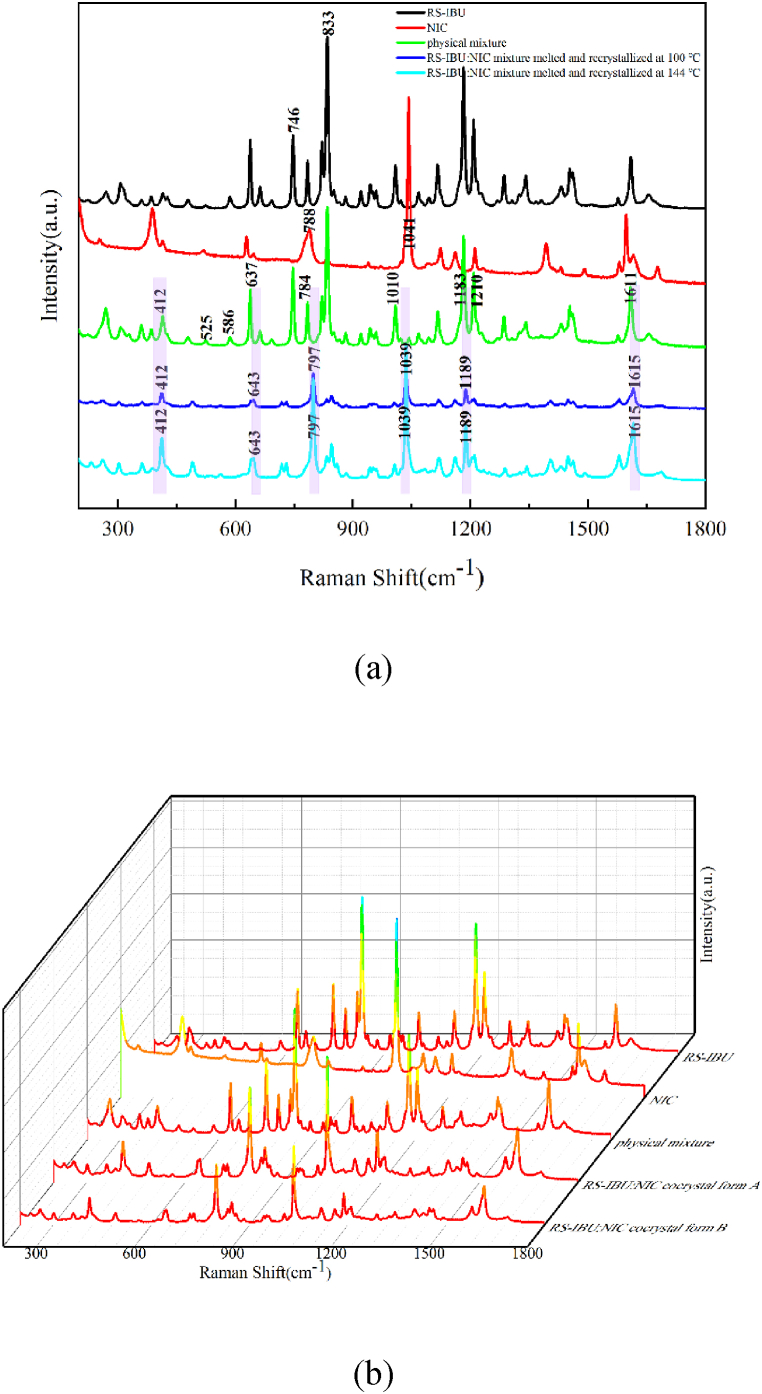
RS-IBU, NIC, physical mixture and RS-IBU: NIC cocrystal form A (a) RS-IBU, NIC, physical mixture and RS-IBU: NIC two cocrystal forms (b) in the range of 200∼1800 cm^−1^ Raman spectra.

The Raman spectra of RS-IBU, NIC, physical mixture, RS-IBU: NIC cocrystal form A and RS-IBU: NIC cocrystal form B in the spectral range of 200∼1800 cm^−1^ were shown in Fig. 8. As shown in Fig. 8 (a), compared with the Raman spectra of the physical mixture, the frequency shift of some characteristic peaks appeared in the RS-IBU: NIC cocrystal form A. RS-IBU has unique characteristic peaks at 746 cm^−1^ and 833 cm^−1^, which was consistent with what Karimi-Jafari et al. [37] reported. Among them, the characteristic peak at 746 cm^−1^ was formed by C=O and aromatic C-H stretching, and the characteristic peak at 833 cm^−1^ was formed by the stretching of aromatic C-H. NIC has unique characteristic peaks at 788 cm^−1^ and 1041 cm^−1^, which was consistent with what Soares et al. [38] reported. Among them, the characteristic peak at 788 cm^−1^ was due to the angular deformation of the NH_2_ group, and the characteristic peak at 1041 cm^−1^ was due to the stretching of C-N-H and pyridine rings. The Raman spectra of RS-IBU: NIC cocrystal form A has unique characteristic peaks at 643 cm^−1^, 797 cm^−1^, 1039 cm^−1^, 1189 cm^−1^ and 1615 cm^−1^, while the physical mixture only has the same characteristic peak at 412 cm^−1^, and there is a certain degree of shift in other characteristic peaks. Specifically, the characteristic peaks of the physical mixture at 525 cm^−1^ and 586 cm^−1^ did not appear in the cocrystal form A, the characteristic peak of the physical mixture at 637 cm^−1^ was blue-shifted to the characteristic peak of the cocrystal form A at 643 cm^−1^, the characteristic peak of the physical mixture at 784 cm^−1^ was blue-shifted to the characteristic peak of the cocrystal form A at 797 cm^−1^, the characteristic peak of the physical mixture at 1010 cm^−1^ was blue-shifted to the characteristic peak of the cocrystal form A at 1039 cm^−1^, the characteristic peaks of the physical mixture at 1183 cm^−1^ and 1210 cm^−1^ were merged into the characteristic peak of the cocrystal form A at 1189 cm^−1^, and the characteristic peak of the physical mixture at 1611 cm^−1^ was blue-shifted to the characteristic peak of the cocrystal form A at 1615 cm^−1^. For ease of observation, in Fig. 8 (a), the above characteristic peaks were covered by light purple rectangular shadow.

As shown in Fig. 8 (b), RS-IBU: NIC cocrystal form A and RS-IBU: NIC cocrystal form B have the same characteristic peaks, which illustrates that Raman spectral technology cannot identify the differences in crystal arrangement between R S-IBU: NIC cocrystal form A and RS-IBU: NIC cocrystal form B. This may be because Raman spectroscopy can characterize intramolecular forces but cannot sensitively characterize intermolecular forces. Therefore, the differences in crystal arrangement between RS-IBU: NIC cocrystal form A and RS-IBU: NIC cocrystal form B may be mainly due to inconsistent intermolecular hydrogen-bonding interactions.

#### 3.3.2 Raman experimental spectra of S (+)-IBU: NIC cocrystal polymorphs

**Fig. 9:**
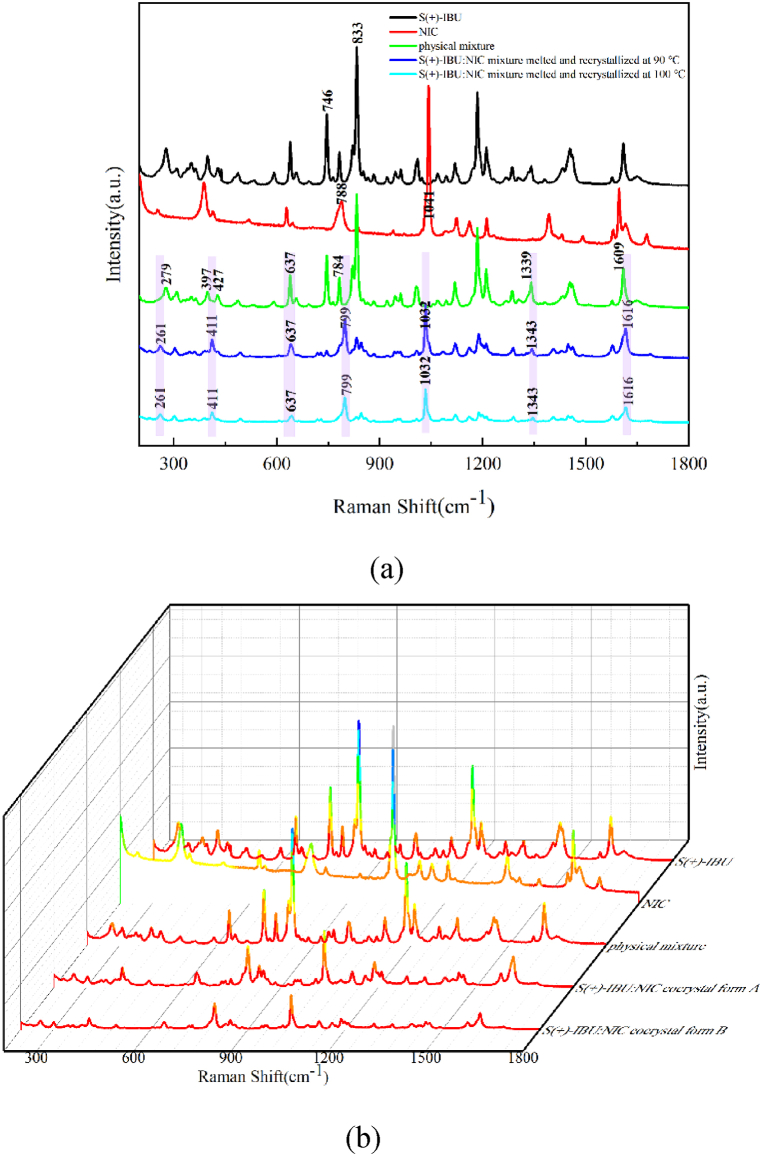
S (+)-IBU, NIC, physical mixture and S (+)-IBU: NIC cocrystal form A (a) S (+)-IBU, NIC, physical mixture and S (+)-IBU: NIC two cocrystal forms (b) in the range of 200∼1800 cm^−1^ Raman spectra.

Raman spectra of S (+)-IBU, NIC, physical mixture, S (+)-IBU: NIC cocrystal form A and S (+)-IBU: NIC cocrystal form B in the spectral range of 200∼1800 cm^−1^, are shown in Fig. 9. As shown in Fig. 9 (a), compared with the Raman spectra of the physical mixture, the frequency shift of some characteristic peaks appears in the S (+)-IBU: NIC cocrystal form A. S (+)-IBU has unique characteristic peaks at 746 cm^−1^ and 833 cm^−1^, which are consistent with the typical characteristic peaks of RS-IBU and have the same formation reason. The Raman spectra of S (+)-IBU: NIC cocrystal form A has unique characteristic peaks at 261 cm^−1^, 411 cm^−1^, 799 cm^−1^, 1032 cm^−1^, 1343 cm^−1^ and 1616 cm^−1^, while the physical mixture only has the same characteristic peak at 637 cm^−1^, and there is a certain degree of shift in other characteristic peaks. For ease of observation, in Fig. 9 (a), the above characteristic peaks were covered by light purple rectangular shadow.

As shown in Fig. 9 (b), S (+)-IBU: NIC cocrystal form A and S (+)-IBU: NIC cocrystal form B have the same characteristic peaks, which illustrates that Raman spectral technology can characterize intramolecular force, while cannot sensitively represent the intermolecular force. Therefore, S (+)-IBU: NIC cocrystal form A and S (+)-IBU: NIC cocrystal form B have different intermolecular hydrogen-bonding interactions, resulting in differences in crystal arrangement, and this difference cannot be identified by Raman spectral technology.

#### 3.3.3 Raman experimental spectra of R (−)-IBU: NIC cocrystal polymorphs

**Fig. 10:**
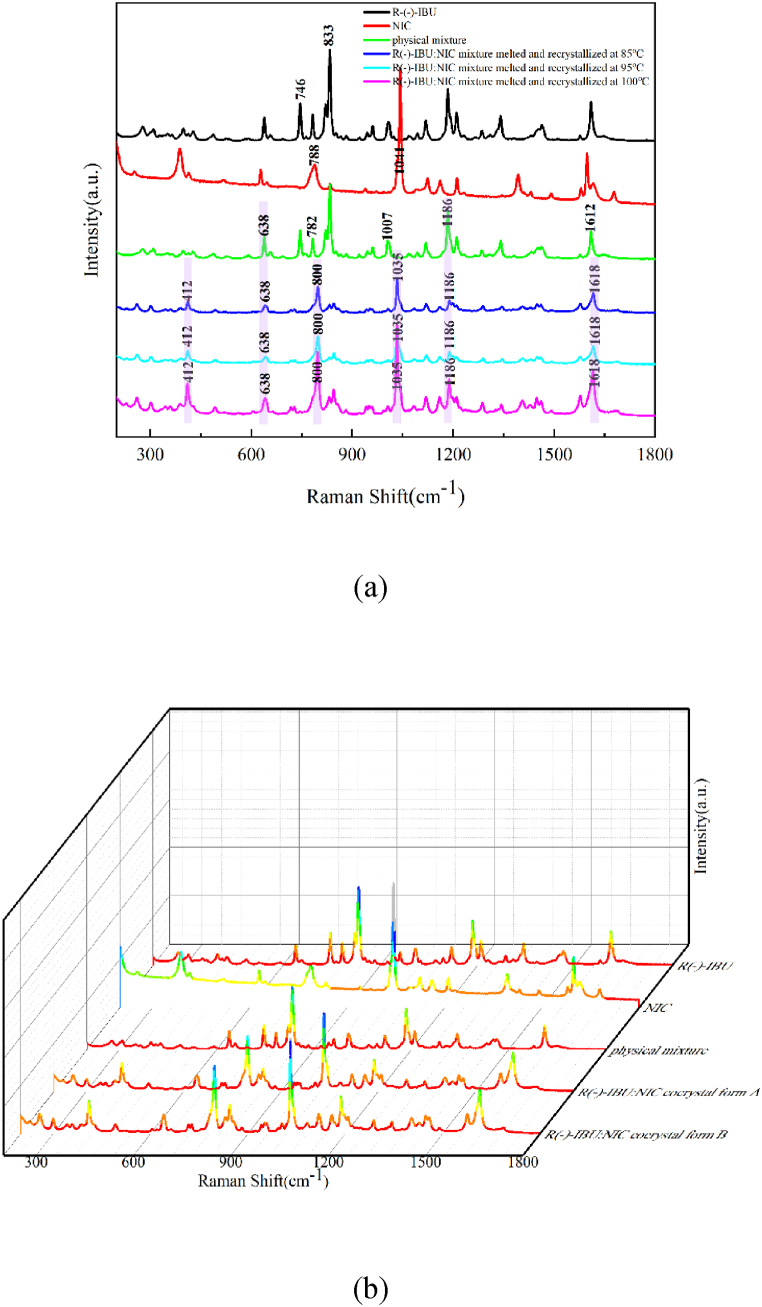
R (−)-IBU, NIC, physical mixture and R (−)-IBU: NIC cocrystal form A (a) R (−)-IBU, NIC, physical mixture and R (−)-IBU: NIC two cocrystal forms (b) in the range of 200∼1800 cm^−1^ Raman spectra.

Raman spectra of R (−)-IBU, NIC, physical mixture, R (−)-IBU: NIC cocrystal form A and R (−)-IBU: NIC cocrystal form B in the spectral range of 200∼1800 cm^−1^ are shown in Fig. 10. As shown in Fig. 10 (a), compared with the Raman spectra of the physical mixture, the frequency shift of some characteristic peaks appears in the R (−)-IBU: NIC cocrystal form A. R (−)-IBU has unique characteristic peaks at 746 cm^−1^ and 833 cm^−1^, which are consistent with the typical characteristic peaks of RS-IBU and S (+)-IBU and have the same formation reason. The Raman spectra of R (−)-IBU: NIC cocrystal form A has unique characteristic peaks at 412 cm^−1^, 800 cm^−1^, 1035 cm^−1^, and 1618 cm^−1^, while the physical mixture has unique peaks at 638 cm^−1^. It has the same characteristic peak at 1186 cm^−1^, and there is a certain degree of shift in other characteristic peaks. For ease of observation, in Fig. 10 (a), the above characteristic peaks were covered by light purple rectangular shadow.

As shown in Fig. 10 (b), R (−)-IBU: NIC cocrystal form A and R (−)-IBU: NIC cocrystal form B have the same characteristic peaks, which illustrates that Raman spectral technology can characterize intramolecular force, while cannot sensitively represent the intermolecular force.

### 3.4 XRD measurement and analysis of RS-IBU: NIC, S (+)-IBU: NIC and R (−)-IBU: NIC cocrystal polymorphs

XRD is a method that uses the diffraction phenomenon of X-rays in crystals to analyze the crystal structure, lattice parameters, crystal defects, content of different crystal phases and internal stress of the material. Its principle is based on Bragg's law, that is, when X-rays pass through When crystals are formed, diffraction phenomena will occur. The diffraction angle is related to the crystal structure and is the main method for quantification of polymorphs [39]. We analyzed in detail the experimentally measured XRD patterns of the cocrystal polymorphs formed by three APIs and NIC in the supplementary material section (Supplementary Figs. S1–S4), and simulated two cocrystal forms of RS-IBU: NIC, the formation of cocrystal polymorphs can be better illustrated by comparison. Among them, Supplementary Fig. S1 showed the XRD patterns of RS-IBU, NIC, physical mixture, RS-IBU: NIC cocrystal form A and cocrystal form B in the range of 2θ from 5 to 50°. It could be seen from the figure that the diffraction peaks of RS-IBU, NIC, physical mixture and the two cocrystals were completely different, and the diffraction peaks of the cocrystal were not a linear superposition of the single-component diffraction peaks. Comparing the XRD patterns of RS-IBU: NIC cocrystal form A and RS-IBU: NIC cocrystal form B, it could be found that the diffraction peaks of the two were very obviously different. Therefore, RS-IBU: NIC cocrystal form A and form B could be completely distinguished through XRD patterns. Among them, Supplementary Fig. S2 showed the XRD patterns of S (+)-IBU, NIC, physical mixture, S (+)-IBU: NIC cocrystal form A and cocrystal form B in the range of 2θ from 5 to 50°. Likewise, a physical mixture is a linear superposition of the diffraction peaks of the starting components, whereas the diffraction peaks of a cocrystal are not a linear superposition of the diffraction peaks of the single components. Supplementary Fig. S3 showed the XRD patterns of R (−)-IBU, NIC, physical mixture, R (−)-IBU: NIC cocrystal form A and cocrystal form B in the range of 2θ from 5 to 50°. Through comparison, it could be found that the absorption peak of R (−)-IBU: NIC cocrystal form A was not observed in the starting components and was completely different from the substance mixture. This may be caused by the intermolecular hydrogen-bonding interactions of R (−)-IBU and NIC, as well as the intermolecular hydrogen-bonding interactions of NIC. To better illustrate the generation of RS-IBU: NIC cocrystal form A and RS-IBU: NIC cocrystal form B, we simulated the XRD patterns of the two crystal forms respectively, as shown in Supplementary Fig. S4. Through comparative analysis, we found that the experimentally measured XRD patterns were consistent with the simulated XRD patterns, which demonstrated the successful preparation of the two cocrystal forms of RS-IBU: NIC.

### 3.5 DFT calculation of RS-IBU: NIC cocrystal polymorphs

We used Gaussian 16 combined with Gaussian-View software to perform quantum chemical theoretical calculations, using B3LYP functional and 6–311++g(d,p) basis set to compare RS-IBU: NIC cocrystal form A and RS-IBU: NIC cocrystal form B [40,41]. The crystal structures of cocrystal polymorphs were geometrically optimized, and the THz and Raman spectra of the two cocrystals were obtained through theoretical calculations. The reliability of the B3LYP functional in ground-state geometry calculations has been extensively evaluated previously [42]. The B3LYP functional was used to calculate the geometric shapes and vibrational frequencies of all molecules, and the calculated wavenumbers were scaled by 0.96 times.

#### 3.5.1 Terahertz simulated spectra of RS-IBU: NIC cocrystal polymorphs

**Fig. 11:**
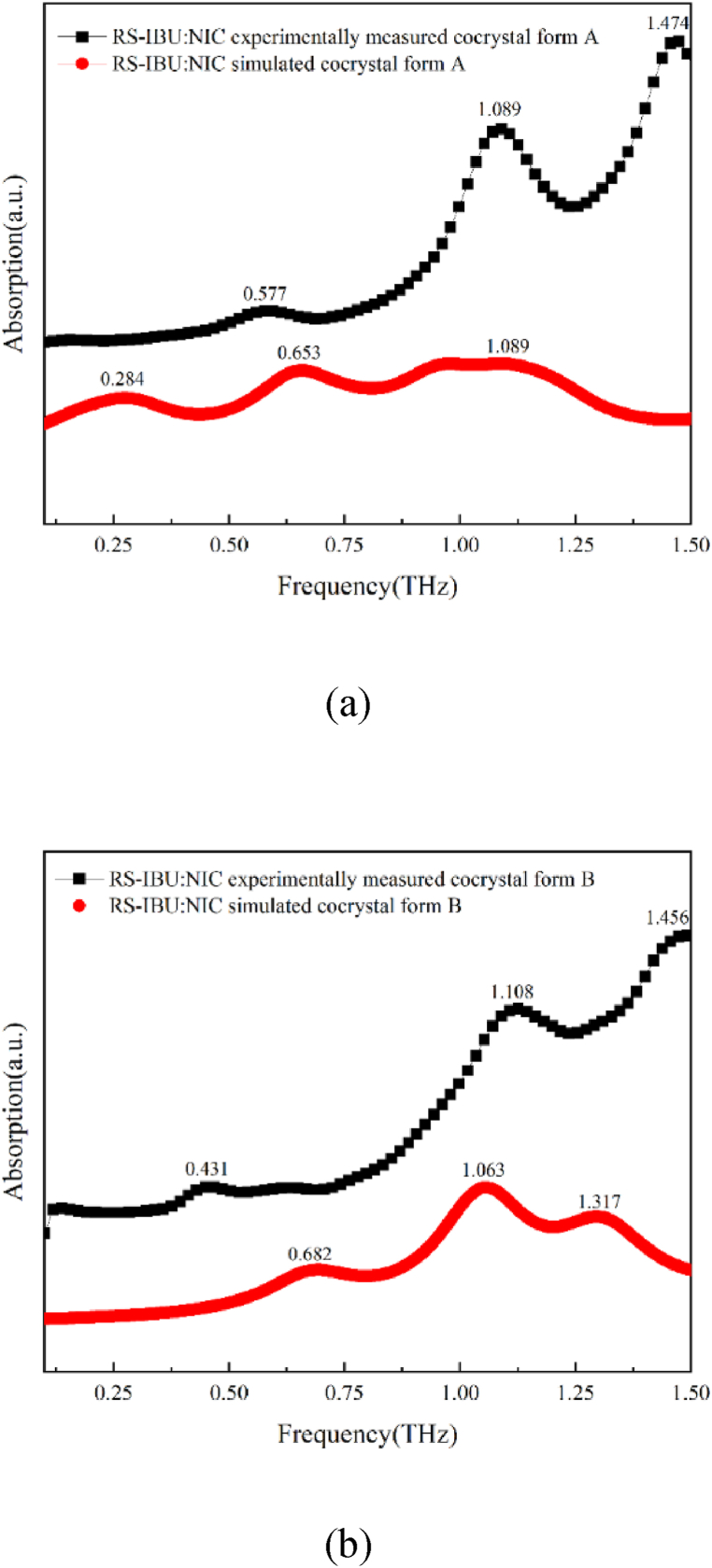
Comparison of experimentally and simulated THz spectra of RS-IBU: NIC cocrystal form A (a) and RS-IBU: NIC cocrystal form B (b).

The THz spectral results of RS-IBU: NIC cocrystal form A and RS-IBU: NIC cocrystal form B measured through theoretical simulation and experiment were shown in Fig. 11. As shown in Fig. 11 (a), the cocrystal form A calculated by DFT has three absorption peaks at 0.284 THz, 0.653 THz and 1.089 THz. By comparing with the experimentally measured spectral results, the spectra of the theoretical form of RS-IBU: NIC cocrystal form A appeared red-shifted. The reason is that the theoretical simulation is calculated at the extreme temperature of absolute zero, and our experimental process is performed at room temperature [[43], [44], [45]].

As shown in Fig. 11 (b), the cocrystal form B calculated by DFT has three absorption peaks at 0.682 THz, 1.063 THz and 1.317 THz. By comparing with the experimentally measured spectral results, one absorption peak of the theoretical spectra of RS-IBU: NIC cocrystal form B has a blue shift, while the other two absorption peaks were relatively close to the experimentally measured results. Based on the analysis of the above terahertz spectrum results, it can be inferred that the structure of the theoretical cocrystal form B is more reasonable than the result of the theoretical cocrystal form A.

#### 3.5.2 Raman simulated spectra of RS-IBU: NIC cocrystal polymorphs

**Fig. 12:**
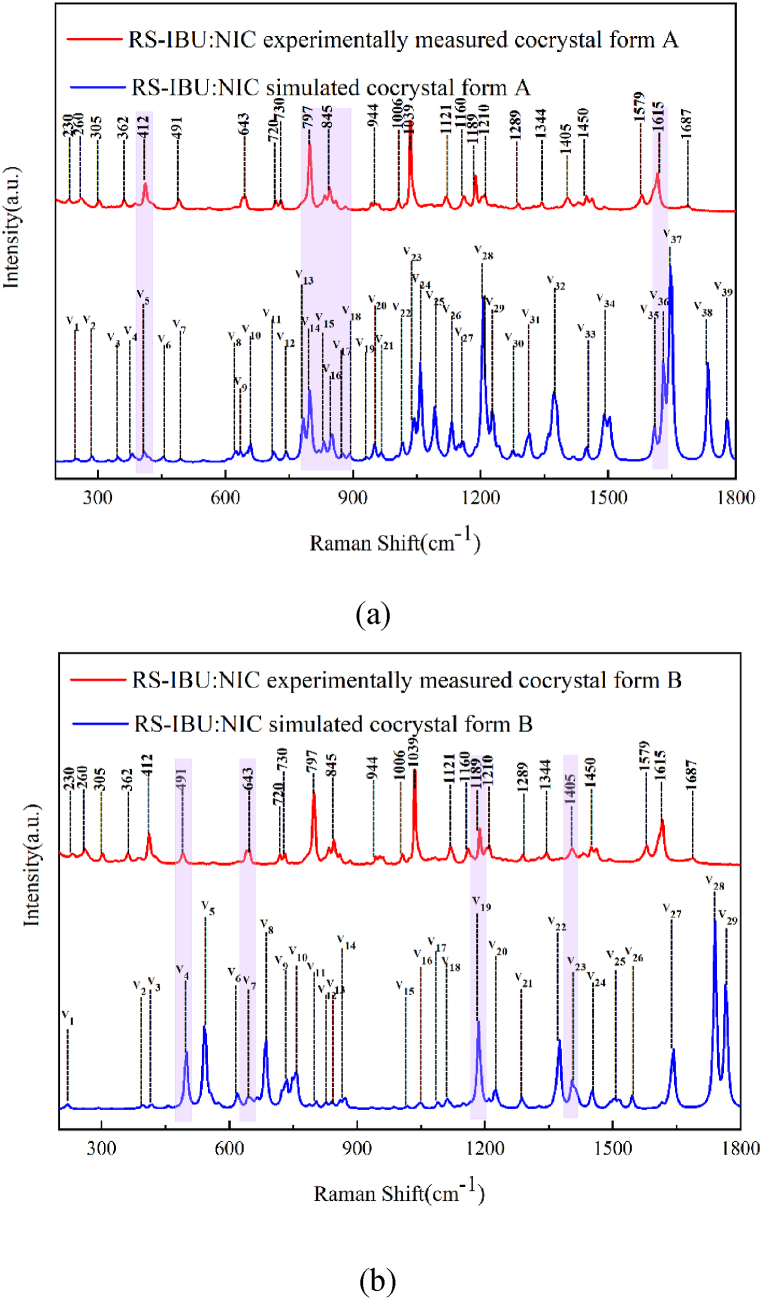
Comparison of experimentally and simulated Raman spectra of RS-IBU: NIC cocrystal form A (a) and RS-IBU: NIC cocrystal form B (b).

Similarly, the Raman spectra of RS-IBU: NIC cocrystal form A and RS-IBU: NIC cocrystal form B were also simulated in a theoretical form, as shown in Fig. 12. It could be seen in Fig. 12 (a), for the RS-IBU: NIC cocrystal form A, the theoretical peaks at 412, 797, and 1615 cm^−1^ were consistent with the three characteristic peaks of the experimental Raman spectra. The above characteristic peaks were covered by light purple rectangular shadow.

As shown in Fig. 12 (b), for the RS-IBU: NIC cocrystal form B, the theoretical peaks at 491, 643, 1189 and 1405 cm^−1^ were consistent with the four characteristic peaks of the experimental Raman spectra (The above characteristic peaks were covered by light purple rectangular shadow). In the theoretical results, only one structural unit of the cocrystal molecular network was calculated, while the Raman spectra measured in the experiment obtained the entire solid-state eutectic molecular network, which might also cause a certain frequency shift. Based on the above analysis of the Raman spectral results, it can be inferred that the structural optimization effect of the theoretical cocrystal form B is better than that of the theoretical cocrystal form A.

#### 3.5.3 Vibrational modes of RS-IBU: NIC cocrystal polymorphs

**Fig. 13:**
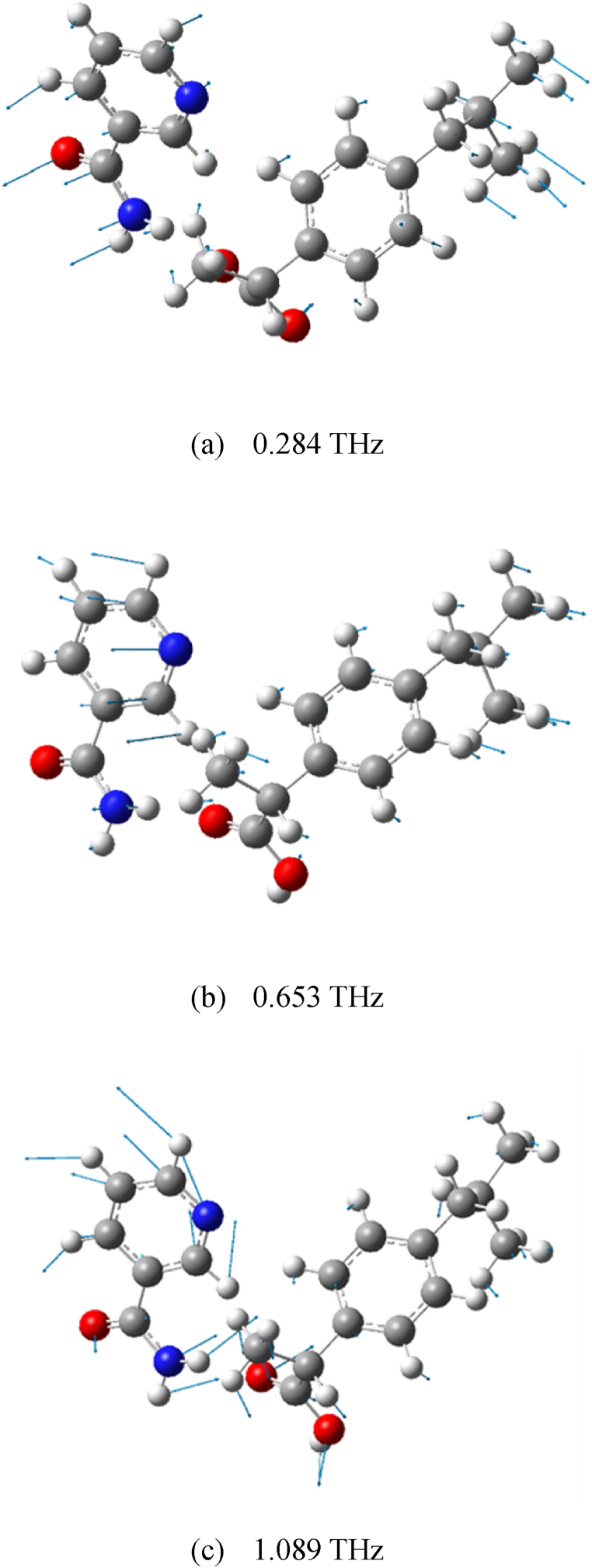
Vibrational modes of RS-IBU: NIC cocrystal form A at 0.284 THz (a), 0.653 THz (b) and 1.089 THz (c).

**Fig. 14:**
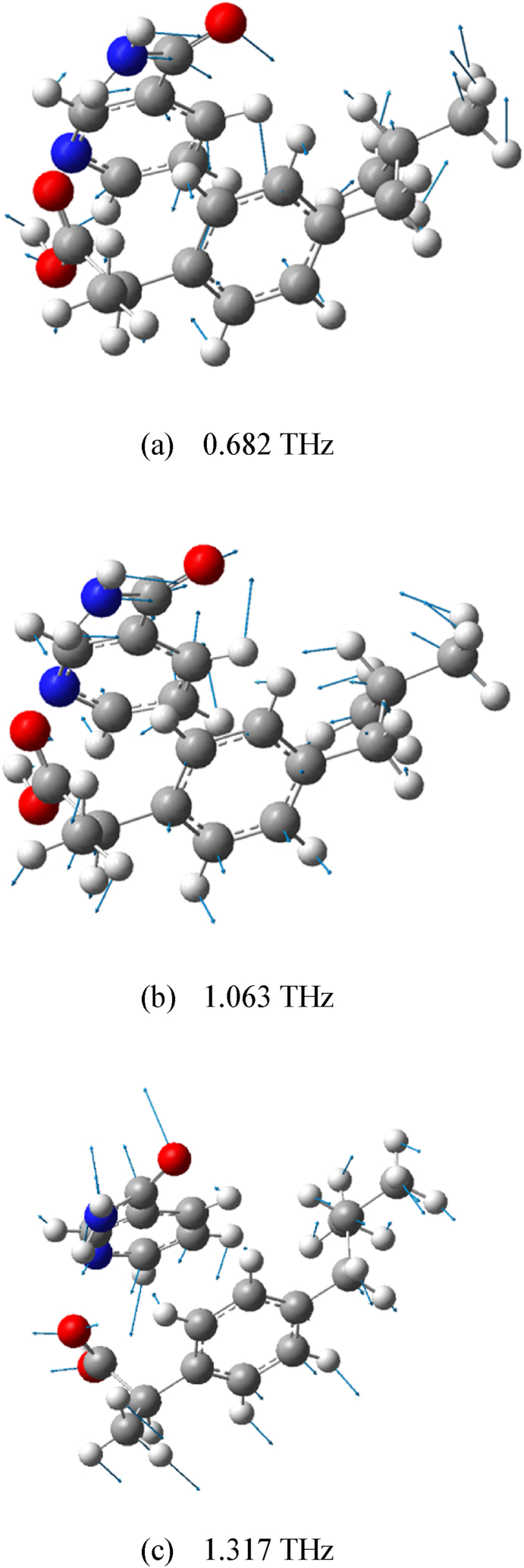
Vibrational modes of RS-IBU: NIC cocrystal form B at 0.682 THz (a), 1.063 THz (b) and 1.317 THz (c).

Gaussian-View is a software program specially designed for the Gaussian function. Its main purpose is to create an input file for the Gaussian function and then display the output calculation results of the Gaussian function in a graphical view. In order to better understand the experimentally measured terahertz spectra, we analyzed the vibrational modes of the two cocrystal forms of RS-IBU: NIC at each absorption peaks through Gaussian-View software, as shown in Fig. 13, Fig. 14. The absorption mechanism of terahertz spectroscopy mainly excites intermolecular vibrations and can be used to sensitively test the hydrogen-bonding effects between various model molecules [[46], [47], [48]]. Different absorption peaks in the terahertz spectra originate from the vibration effects of different groups in the cocrystal structures. It can be observed by assigning vibrational modes that the hydrogen bonders of the cocrystals play an important role in changing its molecular structures and vibrational modes. Different intermolecular hydrogen-bonding interactions lead to the formation of polymorphic forms of the RS-IBU: NIC cocrystal. In Fig. 13, Fig. 14, we use blue arrows to mark the vibration mode assignments of different groups in the crystal structures of RS-IBU: NIC cocrystal form A and cocrystal form B respectively, which are responsible for the main reasons that the absorption peaks in the terahertz spectra are different. Table 1 describes the vibrational mode assignments of RS-IBU: NIC cocrystal form A and RS-IBU: NIC cocrystal form B at different absorption peaks in the terahertz spectra.

**Table 1:** Vibrational mode assignments of RS-IBU: NIC cocrystals in THz spectra.

Cocrystal	Experimental Results	Theoretical Results	Mode Assignment
	f/THz	f/THz	
Form A	0.577	0.284	Out-of-plane bending vibration of RS-IBU molecules, out-of-plane bending vibration of NIC molecules
	1.089	0.653	Out-of-plane bending vibration of RS-IBU molecules, out-of-plane bending vibration of NIC molecules, tensile vibration of benzene rings
	1.474	1.089	In-plane bending vibration of NIC molecules, out-of-plane bending vibration of RS-IBU molecules, torsional vibration of O_31_=C_30_-O_32_-H_33_
Form B	0.431	0.682	In-plane bending vibration of NIC molecules, in-plane bending vibration of RS-IBU molecules, rocking vibration of benzene rings
	1.108	1.063	Out-of-plane bending vibration of NIC molecules, in-plane bending vibration of RS-IBU molecules, rocking vibration of pyridine rings
	1.456	1.317	Out-of-plane bending vibration of NIC molecules, out-of-plane bending vibration of RS-IBU molecules, torsional vibration of O_18_=C_17_-O_19_-H_20_

#### 3.5.4 Hirshfeld surfaces analysis of RS-IBU: NIC cocrystal polymorphs

Hirshfeld surfaces analysis could be color-coded to identify intermolecular interactions, and its two-dimensional fingerprint could visually display the surface area occupied by each intermolecular interactions in the crystals [49]. Among them, $d_{i}$ was the closest distance from the atoms inside the surface to the surface, $d_{e}$ was the closest distance from the atoms outside the surface to the surface. $d_{norm}$ was the distance between $d_{i}$ and $d_{e}$, the closer they were, the stronger the intermolecular force. The red, blue and white colors on the molecular surface were representing negative potential, positive potential and zero potential, respectively. In addition, white represented the weak contact (VdW = $d_{norm}$) of molecules, while red and blue corresponded to the short contact (VdW < $d_{norm}$) and the long contacts (VdW > $d_{norm}$) of molecules, respectively.

Before performing Hirshfeld surfaces analysis, it was necessary to find the CCDC reference number of the target cocrystals in CCDC. By consulting CCDC, we could only obtain the CCDC reference numbers of the two cocrystal forms of RS-IBU: NIC (RS-IBU: NIC cocrystal form A is 678915, and RS-IBU: NIC cocrystal form B is 773196). We knew that the CCDC reference number was obtained through single crystal X-ray diffraction experiments. However, our current experimental conditions could not obtain the CCDC reference numbers of the S (+)-IBU: NIC and R (−)-IBU: NIC cocrystal polymorphs. Therefore, we are temporarily unable to obtain the Hirshfeld surfaces analysis and two-dimensional fingerprint of the S (+)-IBU: NIC and R (−)-IBU: NIC cocrystal polymorphs.

**Fig. 15:**
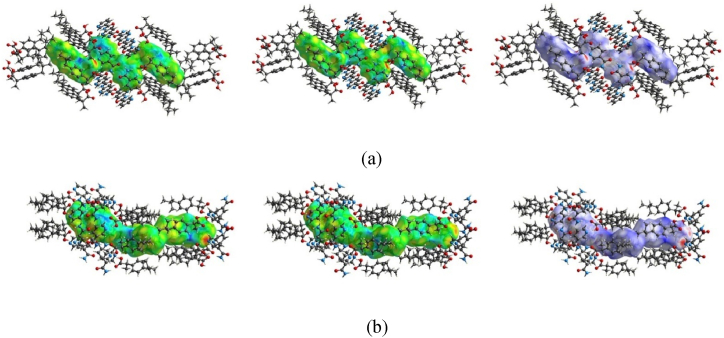
Hirshfeld surfaces analysis of RS-IBU: NIC cocrystal form A (a) and RS-IBU: NIC cocrystal form B (b) in $d_{i}$, $d_{e}$ and $d_{norm}$.

The Hirshfeld surfaces analysis of RS-IBU: NIC cocrystal form A and RS-IBU: NIC cocrystal form B in $d_{i}$, $d_{e}$ and $d_{norm}$ with a norm of 3.8 Å were shown in Fig. 15. It could be seen from Fig. 15 (a), there were two bright red areas on the Hirshfeld surface ($d_{norm}$) of the RS-IBU: NIC cocrystal form A, which respectively represented the hydrogen bonds of intermolecular interactions formed by RS-IBU molecules and NIC molecules (O_21_-H_21_···N_52_ and O_1_-H_1_···N_42_), and the two weak red areas represented the hydrogen bonds formed between NIC molecules (N_51_-H_51A_···O_41_ and N_41_-H_41A_··· O_51_). Among them, $d_{i}$ was mapped to a fixed color scale from 0.6515 to 2.6441 Å, $d_{e}$ was mapped to a fixed color scale from 0.6511 to 2.6472 Å and the $d_{norm}$ surface was mapped to a fixed color scale from −0.7313 to 1.7446 Å, respectively. It could be seen from Fig. 15 (b), there were two bright red areas on the Hirshfeld surfaces analysis ($d_{norm}$) of the RS-IBU: NIC cocrystal form B, which represented the hydrogen bonds (O_6_-H_6A_··· N_2_ and O_4_-H_4A_···N_4_), while the two weak red areas represented the hydrogen bonds formed between NIC molecules (O_2_···H_1A_-N_1_ and N_3_-H_3A_···O_1_) respectively. Among them, $d_{i}$ was mapped to a fixed color scale from 0.6398 to 2.6578 Å, $d_{e}$ was mapped to a fixed color scale from 0.6406 to 2.6492 Å and the $d_{norm}$ surface was mapped to a fixed color scale from −0.7494 to 1.7415 Å, respectively. In addition, the blue areas ($d_{norm}$) of the Hirshfeld surfaces analysis represented the H···H interactions of the RS-IBU: NIC cocrystal polymorphism.

**Fig. 16:**
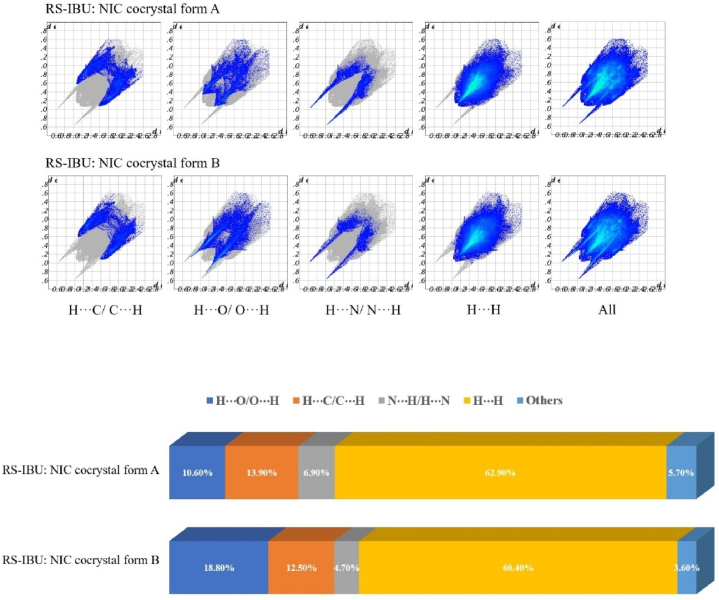
Two-dimensional fingerprint of RS-IBU: NIC cocrystal form A and RS-IBU: NIC cocrystal form B.

The two-dimensional fingerprint of RS-IBU: NIC cocrystal form A and RS-IBU: NIC cocrystal form B were shown in Fig. 16. It could be seen from the figure, H···H intermolecular interactions accounted for the largest proportion in the two-dimensional fingerprint, accounting for 62.90 % and 60.40 % in RS-IBU: NIC cocrystal form A and RS-IBU: NIC cocrystal form B respectively. In RS-IBU: NIC cocrystal form A, the proportion of O-H···N intermolecular hydrogen bonds between RS-IBU molecules and NIC molecules was 6.90 %, while the proportion of N-H···O intermolecular hydrogen bonds in NIC molecules was 10.60 %. In RS-IBU: NIC cocrystal form B, the proportion of O-H···N intermolecular hydrogen bonds between RS-IBU and NIC molecules was 4.70 %, while the proportion of N-H···O intermolecular hydrogen bonds in NIC molecules was 18.80 %. The two sharp peaks in the two-dimensional fingerprint represented hydrogen bonds of intermolecular interactions. Among them, the sharp peaks in the upper left corner ($d_{i}$ <$d_{e}$) represented the point of the hydrogen-bonding donors on the Hirshfeld surfaces, and the sharp peaks in the lower right corner ($d_{i}$ >$d_{e}$) represented the point of the hydrogen-bonding acceptors on the Hirshfeld surfaces. The proportions of H···C/C···H in the two cocrystal forms were 13.90 % and 12.50 %, respectively. Other intermolecular interactions, such as van der Waals forces and π-π stacking, accounted for 5.70 % and 3.60 % respectively.

## 4 Conclusion

In this paper, RS-IBU: NIC, S (+)-IBU: NIC, and R (−)-IBU: NIC cocrystal polymorphs were prepared in a stoichiometric ratio of 1:1 by solution evaporation crystallization, solvent dripping, and melt recrystallization. THz and Raman spectral technology combined with DFT simulation to study the formation process of pharmaceutical cocrystals could effectively analyze the intermolecular and intramolecular interactions and vibrational modes of the cocrystal formed by three kinds of IBU with NIC, making the detection results more accurate. XRD experiments also assisted in illustrating the successful preparation of cocrystal polymorphs. Based on the theoretical simulated results, we assigned vibration modes of the two cocrystal forms of RS-IBU: NIC and found that they were closely related to the corresponding hydrogen bonds, which further proved the bonding modes of hydrogen bonds and the role of hydrogen bonds in the formation of cocrystals. In addition, we discussed a solubility test experiment, and the results confirmed that the formation of cocrystals could indeed improve the solubility of RS-IBU molecules.

This study fills the key gap in the current research on R (−)-IBU: NIC cocrystal polymorphs. Through this work, we have obtained a large amount of spectral information and simulation data. These research results are helpful to provide new ideas and data support for the preparation and analysis of pharmaceutical cocrystals. Therefore, this work is of great significance for the practical application of pharmaceutical cocrystals.

## CRediT authorship contribution statement

**Yaqi Jing:** Software, Formal analysis, Data curation. **Qiuhui Zhao:** Validation, Software, Formal analysis, Data curation. **Jiale Zhang:** Software, Methodology, Formal analysis, Data curation. **Jiadan Xue:** Supervision, Methodology, Funding acquisition. **Jianjun Liu:** Methodology, Investigation, Formal analysis, Data curation. **Jianyuan Qin:** Software, Investigation, Funding acquisition, Conceptualization. **Zhi Hong:** Supervision, Methodology, Investigation. **Yong Du:** Writing – review & editing, Supervision, Project administration, Methodology, Investigation, Funding acquisition.

## Data availability

Data will be made available on request.

## Funding

This research was funded by the 10.13039/501100001809National Natural Science Foundation of China (10.13039/501100001809NSFC, Grant Nos. 62275238, 21973082, 32371983) and 10.13039/501100004731Zhejiang Provincial Natural Science Foundation of China (No. LY19B050003).

## Declaration of competing interest

The authors declare that they have no known competing financial interests or personal relationships that could have appeared to influence the work reported in this paper.
