# Supplementary material for: RS, S (+) - and R (−)-ibuprofen cocrystal polymorphs: Vibrational spectra, XRD measurement and DFT calculation studies
Source: Heliyon. 2025 Jan 20;11(3):e41986. doi: 10.1016/j.heliyon.2025.e41986 (PMC11804694; doi:10.1016/j.heliyon.2025.e41986)
Supplement: Multimedia component 1 [file mmc1.docx]

**Supporting Information**

**RS, S (+) - and R (-)-ibuprofen cocrystal polymorphs: vibrational spectra, XRD measurement and DFT calculation studies**

Yaqi Jing ^a^, Qiuhui Zhao ^a^, Jiale Zhang ^a^, Jiadan Xue ^b^, Jianjun Liu ^a^, Jianyuan Qin ^a^, Zhi Hong ^a^, Yong Du ^a, *^

*^a^Centre for THz Research, China Jiliang University, Hangzhou 310018, China*

*^b^Department of Chemistry, Zhejiang Sci-Tech University, Hangzhou 310018, China*

**Corresponding author. Tel/fax: +86-571-86875618*

*E-mail addresses:* [*yongdu@cjlu.edu.cn*](mailto:yongdu@cjlu.edu.cn)

**Contents**

[Fig. S1. RS-IBU, NIC, physical mixture and RS-IBU: NIC cocrystal form A (a) RS-IBU, NIC, physical mixture and RS-IBU: NIC cocrystal form B (b) in the range of 5~50 ° XRD pattern. 2](#_Toc185498426)

[Fig. S2. S (+)-IBU, NIC, physical mixture and S (+)-IBU: NIC cocrystal form A (a) S (+)-IBU, NIC, physical mixture and S (+)-IBU: NIC cocrystal form B (b) in the range of 5~50 ° XRD pattern. 3](#_Toc185498427)

[Fig. S3. R (-)-IBU, NIC, physical mixture and R (-)-IBU: NIC cocrystal form A (a) R (-)-IBU, NIC, physical mixture and R (-)-IBU: NIC cocrystal form B (b) in the range of 5~50 ° XRD pattern. 4](#_Toc185498428)

[Fig. S4. RS-IBU: NIC experimentally measured cocrystal form A (a), RS-IBU: NIC simulated cocrystal form A (b), RS-IBU: NIC experimentally measured cocrystal form B (c) and RS-IBU: NIC simulated cocrystal form B (d) in the range of 5~50 °XRD pattern. 5](#_Toc185498429)

[Table S1 Crystallographic data for RS-IBU: NIC cocrystal polymorphs. 6](#_Toc185498430)


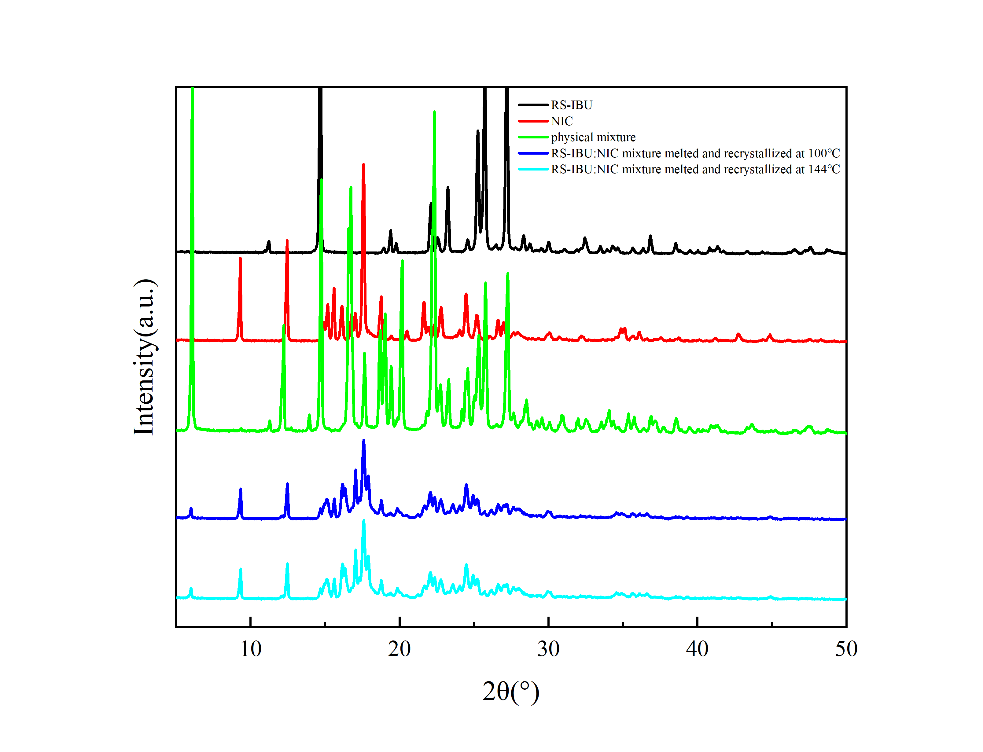


(a)


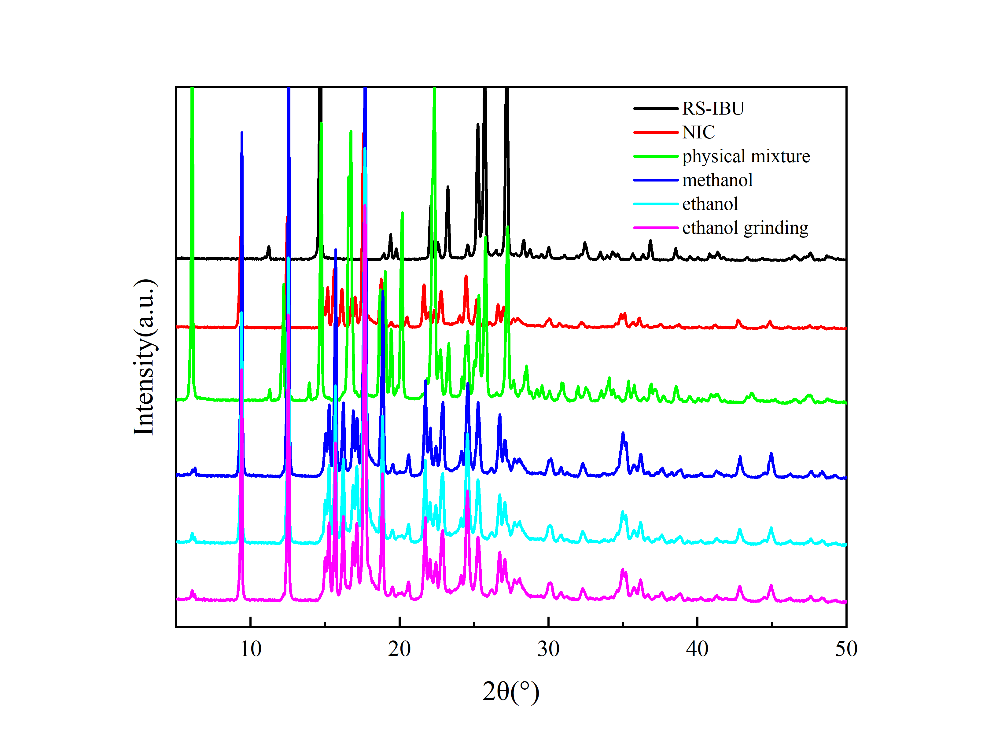


(b)

Fig. S1. RS-IBU, NIC, physical mixture and RS-IBU: NIC cocrystal form A (a) RS-IBU, NIC, physical mixture and RS-IBU: NIC cocrystal form B (b) in the range of 5~50 ° XRD pattern.


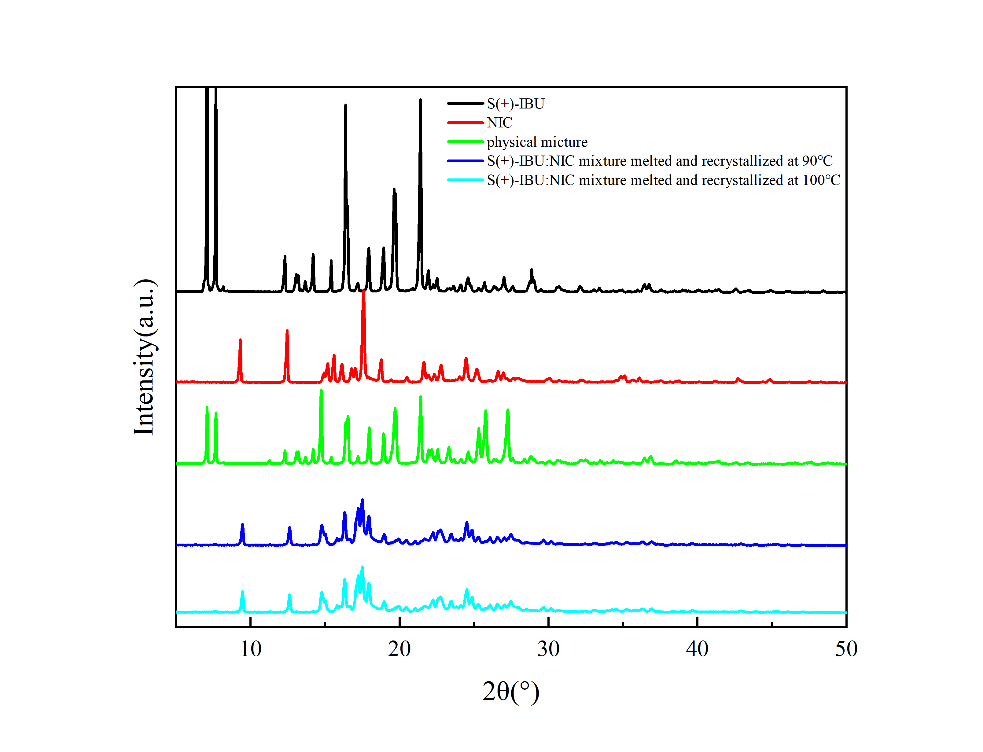


(a)


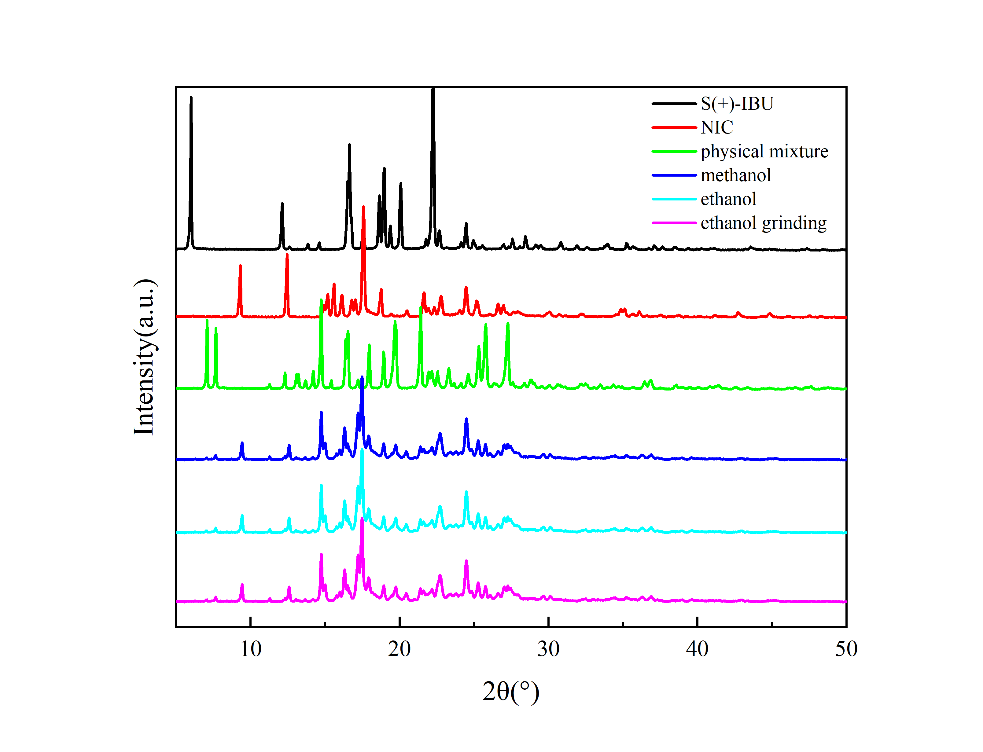


(b)

Fig. S2. S (+)-IBU, NIC, physical mixture and S (+)-IBU: NIC cocrystal form A (a) S (+)-IBU, NIC, physical mixture and S (+)-IBU: NIC cocrystal form B (b) in the range of 5~50 ° XRD pattern.


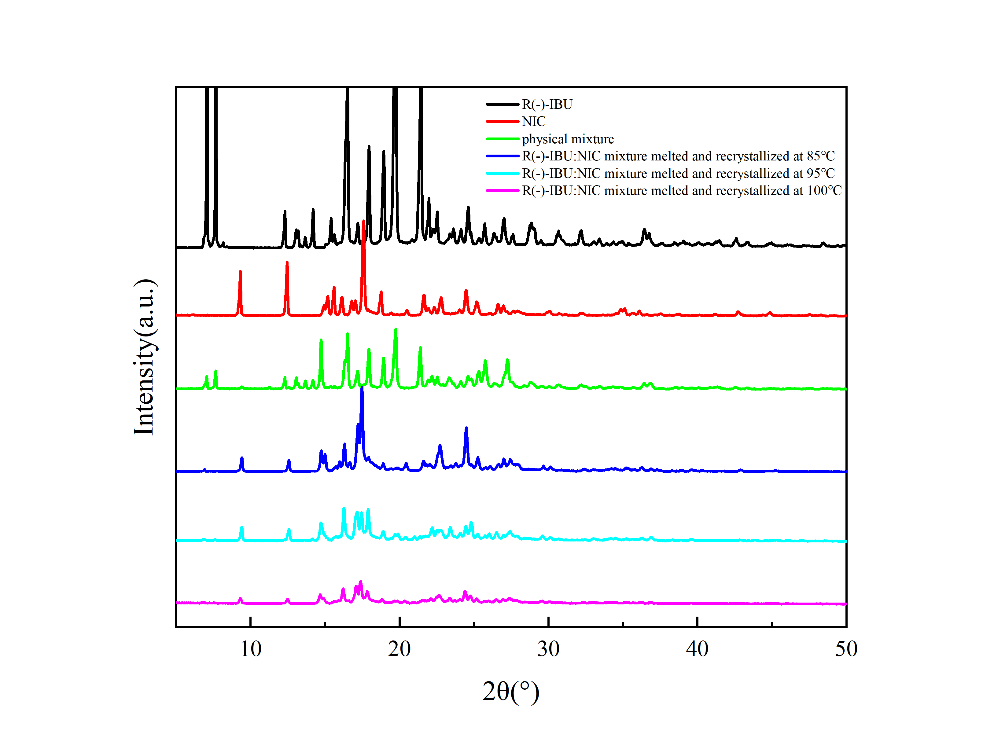


(a)


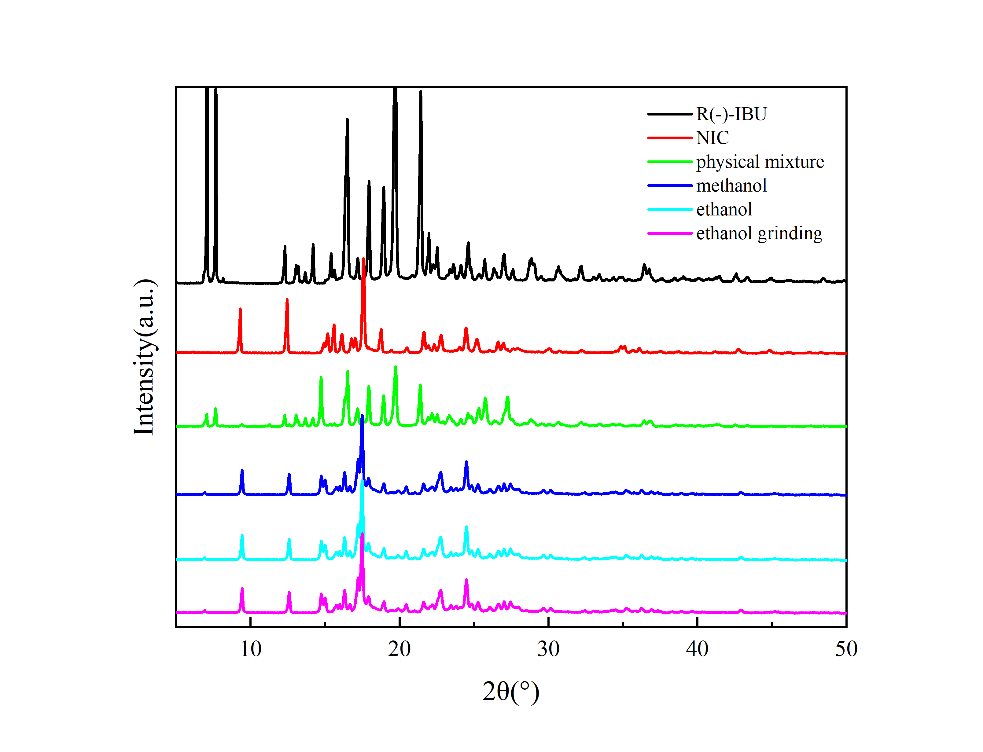


(b)

Fig. S3. R (-)-IBU, NIC, physical mixture and R (-)-IBU: NIC cocrystal form A (a) R (-)-IBU, NIC, physical mixture and R (-)-IBU: NIC cocrystal form B (b) in the range of 5~50 ° XRD pattern.


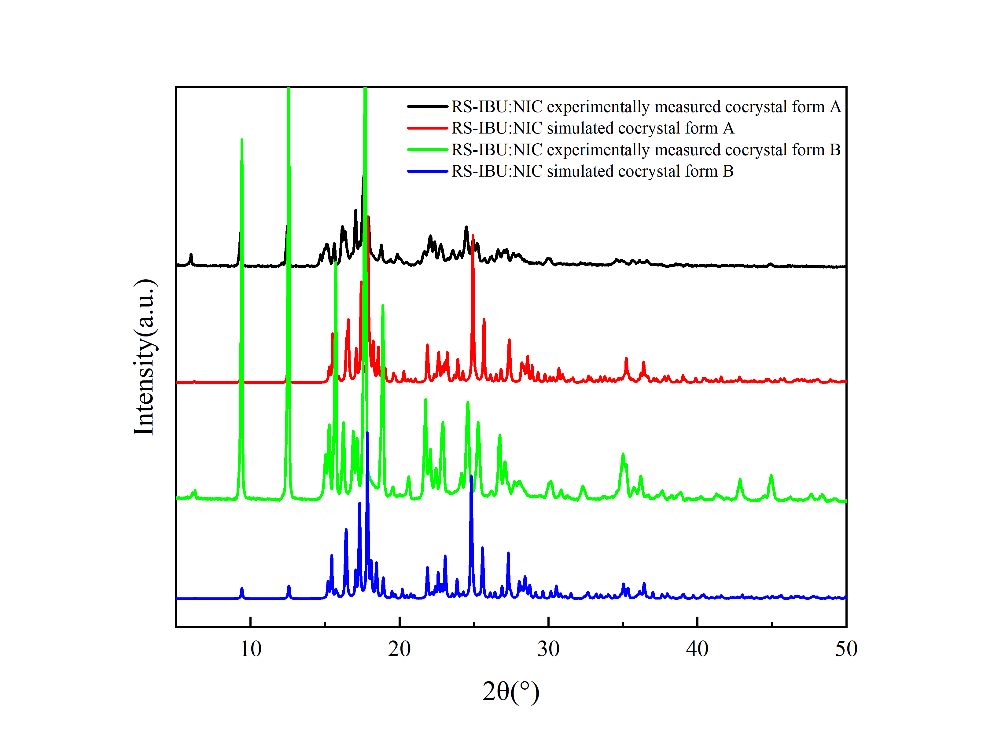


Fig. S4. RS-IBU: NIC experimentally measured cocrystal form A (a), RS-IBU: NIC simulated cocrystal form A (b), RS-IBU: NIC experimentally measured cocrystal form B (c) and RS-IBU: NIC simulated cocrystal form B (d) in the range of 5~50 °XRD pattern.

Table S1 Crystallographic data for RS-IBU: NIC cocrystal polymorphs.

|  | Form A ^[1]^ | Form B ^[2]^ |
| --- | --- | --- |
| Molecular Formula | C_19_H_24_N_2_O_3_ | C_19_H_24_N_2_O_3_ |
| Molecular Weight/g mol^-1^ | 328.4 | 328.4 |
| Crystal system | Orthorhombic | Orthorhombic |
| Space group | $Pca2_{1}$(29) | $Pca2_{1}$(29) |
| a/Å | 11.652(2) | 11.7129(16) |
| b/Å | 5.4473(11) | 5.4915(8) |
| c/Å | 56.607(11) | 56.289(7) |
| α/° | 90.00 | 90.00 |
| β/° | 90.00 | 90.00 |
| γ/° | 90.00 | 90.00 |
| Z | 8 | 0 |
| Z' | 0 | 0 |
| V/Å^3^ | 3592.96 | 3620.59 |
| CCDC number | 678915 | 773196 |

(Note: [1] Crystallographic data for RS-IBU: NIC cocrystal form A were obtained from the Cambridge Crystallographic Data Center and its CCDC reference number was 678915. [2] Crystallographic data for RS-IBU: NIC cocrystal form B were obtained from the Cambridge Crystallographic Data Center and its CCDC reference number was 773196)
